# High-dose polystyrene nanoparticles trigger aberrant activation of the MAPK pathway in spinal cord and pain hypersensitivity

**DOI:** 10.1186/s12951-026-04186-8

**Published:** 2026-03-02

**Authors:** Yuan Yin, Panyang Gu, Hanyu Jiang, Yumei Yang, Shujun Wang, Fei Yuan, Wenrui Zhong, Miao Chen, Meichun Deng

**Affiliations:** 1 Department of Biochemistry and Molecular Biology & Hunan Province Key Laboratory of Basic and Applied Hematology, School of Life Sciences, Central South University, Changsha, 410013 Hunan China; 2 Hunan Key Laboratory of Animal Models for Human Diseases, Hunan Key Laboratory of Medical Genetics, School of Life Sciences, Central South University, Changsha, China

**Keywords:** Polystyrene nanoplastics, Pain, Mitogen-Activated protein kinase, PLX5622, Microglia

## Abstract

The widespread use of plastic products has led to a serious environmental problem, with nanoplastics ubiquitously contaminating the environment and sustaining human exposure, yet the impacts of nanoplastics on human health remain poorly understood. In this study, based on preliminary epidemiological investigations, we found that abnormal pain perception exists in populations chronically exposed to manufacturing environments that mainly produce polystyrene plastics. Further mechanistic studies demonstrated that high-dose polystyrene nanoparticles (PS NPs) induce pain hypersensitivity and elucidated their molecular underpinnings. Upon high-dose PS NPs exposure, microglia in the spinal dorsal horn internalized a fraction of the PS NPs, which were subsequently found to bind to mitogen-activated-protein-kinases (MAPK) pathway components (ERK, JNK, and p38). Molecular dynamics simulations further suggested that this binding could induce conformational alterations in the MAPK components, potentially enhancing the flexibility of their phosphorylation sites (Thr-X-Tyr) and thereby facilitating activation by upstream kinases. As a canonical inflammatory and pain-associated pathway, MAPK activation elevates neuroinflammatory cascades in the spinal dorsal horn, driving neuronal hyperexcitability and, consequently, pain hypersensitivity. Notably, the PS NPs-induced hypersensitivity was reversed by microglial depletion (PLX5622) and inhibition of the MAPK pathway. Collectively, our findings delineate PS NPs-triggered sensory pathophysiology and establish a proof-of-concept mechanistic nexus between environmental pollutants and aberrant somatosensation.

**Graphical abstract:**

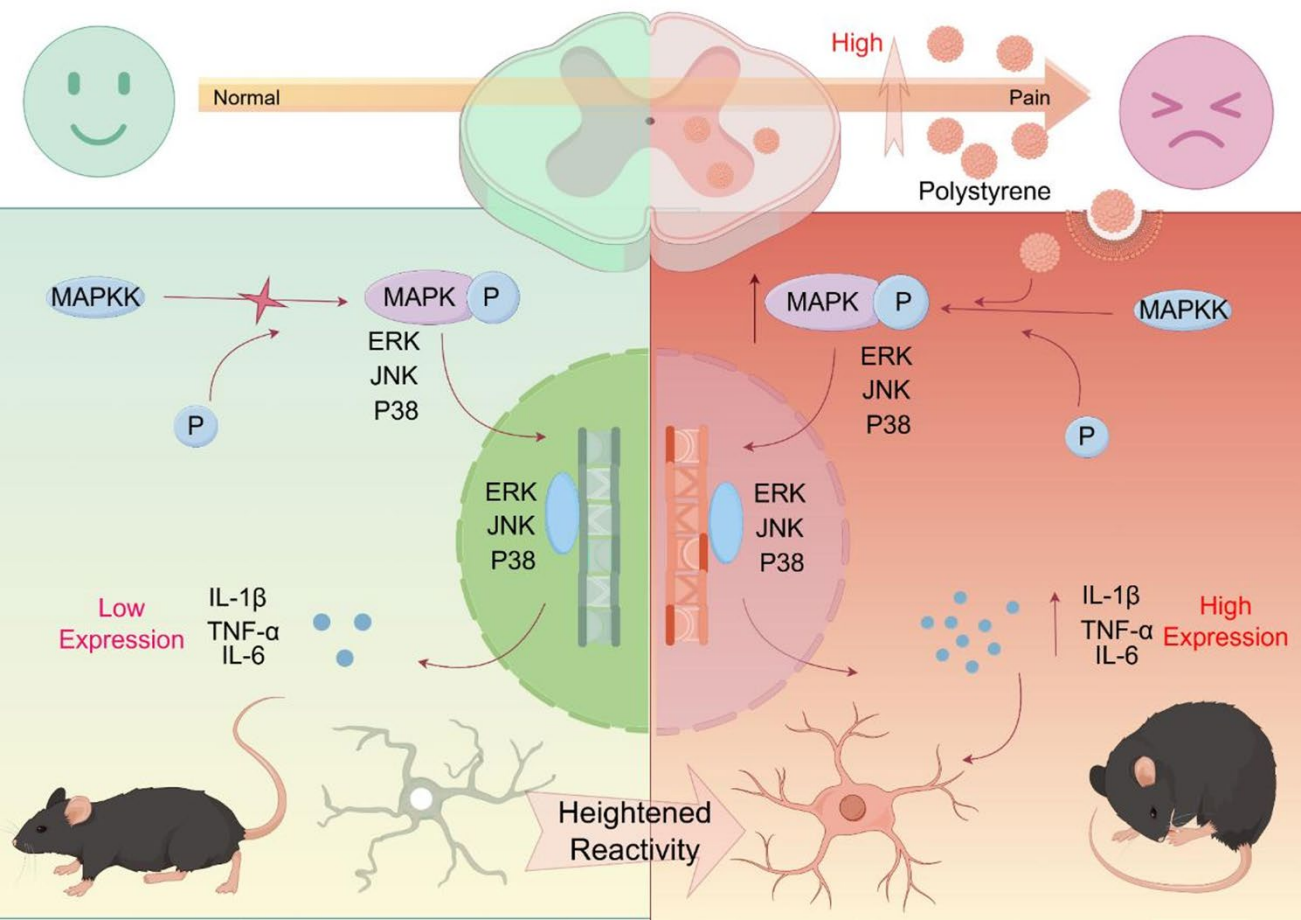

**Supplementary Information:**

The online version contains supplementary material available at 10.1186/s12951-026-04186-8.

## Introduction

The extensive use of plastic products has caused severe environmental pollution, exceeding the ecological assimilation capacity of environmental systems. The persistent, non-biodegradable plastic waste is projected to accumulate to 7 billion metric tons, with over 5 billion tons of plastic waste will have been placed in the environment [1]. Among these plastic products, due to its excellent physical properties, low cost, and ease of processing, polystyrene (PS) is one of the top five mass-produced plastics with an annual output of over 17 million tons [2], and its sustainability has always been a challenge. It has been estimated that between 2.13 × 10^5^ and 9.79 × 10⁷ microplastic particles (approximately 0.01 to 10.85 g) are released during the processing of each ton of plastic [3]. Recent work detected nanoplastic concentrations of approximately 1.5–32.0 mg m⁻³ throughout the water column in the North Atlantic, with PS identified as a primary component [4]. Particularly, estimates suggest that industrial processes like extrusion and spraying release inhalable PS particles into air, where concentrations are 100-1,000 times higher than natural settings [5].

Epidemiological studies have demonstrated that workers exposed to such high levels of PS microplastics are more susceptible to chronic obstructive pulmonary disease (COPD), with up to a 2.5-fold increase in incidence [6, 7]. PS microplastics have been found in lung, spleen, kidney, placenta, and brain, as well as in breast milk and blood of humans [8]. Critically, nanoscale PS particles under 100 nm can cross the blood-brain barrier and accumulate in the brain, where the concentration reaches up to 4917 µg/g [9]. At such high concentrations in the brain, PS nanoparticles (NPs) can trigger neuroinflammation, impair synaptic plasticity, downregulate neurodevelopment-related gene expression, and disrupt glymphatic system function, ultimately leading to neurotoxic effects including cognitive deficits, anxiety/depression-like behaviors, and social dysfunction [10–12]. In addition, recent work has revealed that the benzene rings of PS NPs can bind to TDP-43 proteins upon cellular internalization, altering their phase separation and aggregation behavior—a pathological process that ultimately contributes to neurodegenerative diseases such as amyotrophic lateral sclerosis^6^. Pain responses, as a complex pathological reaction mediated by neural pathways, is an important clinical manifestation of such nerve injury [13]. Consequently, a better understanding of the neurotoxic effects of PS NPs, particularly whether occupational exposure to high concentrations in PS manufacturing facilities induces pain responses, is essential for evaluating their long-term health impacts and guiding the development of safer plastic alternatives. However, the health impacts of high-dose PS NPs exposure on the human nervous system remain unknown, necessitating further research to clarify risks and guide safety regulations.

Over recent decades, microglia, the resident macrophages of the CNS (Central Nervous System), have been extensively studied and causally linked to somatosensory dysfunction and pain-related behaviors [14]. Recently, microglia replacement therapy has been demonstrated to correct disease-causing genetic mutations and effectively block disease progression in both mouse models and human patients [15]. Significantly, previous research demonstrated that PS NPs induce brain inflammation in mice, marked by an increase in disease-associated microglia (DAM) and neuronal degeneration [16]. Mitogen-activated protein kinases (MAPKs), including extracellular signal-regulated kinase (ERK), p38 MAPK, and c-Jun N-terminal kinase (JNK), critically regulate pro-inflammatory cytokine expression during increased microglial reactivity [17], thereby driving pain progression [18]. Prior studies confirm PS NPs exposure activates ERK/p38 in murine hepatocytes and cortical neurons [19]. As the primary relay station for nociceptive signals, the spinal dorsal horn contains neural circuits critical for modulating pain transmission and contributing to chronic pain states [20]. However, the impact of PS NPs on spinal microglial reactivity via the MAPK signaling pathway remains critically undefined. Elucidating the precise molecular cascades underlying PS NPs-induced MAPK activation is imperative to decode neuroimmune mechanisms in pain pathogenesis.

Herein, we conducted an epidemiological survey of workers in plastic factories primarily manufacturing PS plastics. Data revealed high prevalence of hyperalgesia and excessive pain-related fear among the workforce. Subsequent rodent studies demonstrated that short-term high-dose PS NP exposure induced hyperalgesia in mice as early as post-exposure day 3—prior to the reported onset of other neurotoxic effects. This temporal precedence suggests that hyperalgesia may represent an early indicator within the spectrum of PS NP-induced neurological alterations, the potential contribution of which to later-emerging emotional or cognitive abnormalities warrants further investigation. Mechanistic investigations confirmed that PS NPs induce morphological and functional changes in microglia within the spinal dorsal horn, driving neuronal hyperexcitability. RNA sequencing combined with pull-down assays verified direct MAPK pathway activation by PS-NPs, while molecular dynamics simulations elucidated structural bases of PS NP-induced MAPK phosphorylation. Furthermore, microglia replacement therapy and MAPK pathway inhibition effectively suppress the development and progression of PS NPs-related pain pathologies. Collectively, our findings establish novel evidence linking PS-NPs to chronic pain pathogenesis. This research specifically deciphers direct neurotoxicity mechanisms mediated through PS NP-protein interactions, delivering pivotal insights into exposure consequences.

## Experimental section

### Materials and instruments

All experimental protocols involving mice were approved by the Animal Care and Use Committee of Central South University (CSU-2023-0121). All procedures involving vertebrate animals strictly adhered to the approved protocols in accordance with the institutional guidelines. The mice were group-housed under a standard 12-hour light/dark cycle and provided ad libitum access to food and water. Adult male C57BL6/J mice (6–8 weeks) were used in all animal experiments. A previous study reported that the global average human intake of microplastics ranges from 0.1 to 5 g per week [21, 22], approximately equivalent to 0.2–11.9 mg/kg per day. Based on the body surface area (BSA) normalization method, the human-to-mouse conversion factor is 12, resulting in an estimated microplastic exposure range of 2.4–142.8 mg/kg/day for mice [23]. After characterization and quantification, all PS NPs were administered via oral gavage unless otherwise specified.

All chemicals were used in their raw forms unless specified otherwise. Tetramethylammonium hydroxide (TMAH) was purchased from TCI Co. (Shanghai, China). Proteinase K was purchased from Macklin (Shanghai, China). Benzonase and trypsin were purchased from the Aladdin Co. (Shanghai, China). Lipase was purchased from Sigma-Aldrich (St. Louis, MO, USA). PLX5622 and SB203580 were purchased from Aladdin (Shanghai, China), U0126 from Beyotime Biotechnology (Shanghai, China), and SP600125 from Macklin (Jiangsu, China).

Scanning electron microscopy (SEM) was performed using a Tescan MIRA 3 XMU SEM. The zeta potentials of nanoparticles were measured using a Nano-ZS90 Zetasizer (Malvern, UK). Fourier transform infrared (FT-IR) spectra were obtained using a Fourier transform near-infrared spectrophotometer (Nicolet 6700, MA, USA) with a spectral width of 4000 –400 cm^− 1^. Raman signals were obtained using a laser confocal Raman spectrometer (inVia Reflex, Renishaw, UK) equipped with a 785 nm excitation laser and a microscope (50 × long-focus objective lens) through the optical fiber. Fluorescence stability was determined by measuring the fluorescence intensity of 50 nm F-PS NPs using a Hitachi Fluorescence Spectrophotometer F-4600. Milli-Q water was obtained using a Milli-Q Integral System (Millipore, 18.2 MΩ·cm at 25 °C).

### PSQ-C pain threshold scale

Pain sensitivity was assessed using the PSQ, a clinically useful and valid self-report measure based on pain intensity ratings in daily life situations, to identify patients at risk of severe acute postoperative pain. The PSQ-C is the Mandarin Chinese version of the PSQ that has been validated for assessing pain sensitivity in Chinese populations [24]. The PSQ-C includes 17 items rated on a numerical scale (NRS) from 0 (no pain at all) to 10 (worst pain imaginable) [25]. Simultaneously, the overall pain perception experience (NRS Score) over the past month was assessed as the initial evaluation, and the total PSQ score was calculated as the average rating of all items except the three non-painful items. An average score > 6 was considered indicative of pain sensitivity.

### I-NP4 questionnaire

The I-DN4 scale (Self-administered Version of DN4), also known as the short form of the DN4, is derived from the self-reported portion of the DN4 scale and is one of the most commonly used diagnostic scales for neuropathic pain (NP). As the DN4 scale includes three physical examination-related questions that patients cannot complete independently, the I-DN4 scale was developed. The I-DN4 consists of seven self-reported questions on burning pain, cold pain, electric shock-like pain, tingling, pinprick pain, numbness, and itching. Each “yes” answer is scored 1 point, and “no” is scored 0 points. A total score ≥ 3 indicates the presence of NP components [26].

### PCS questionnaire

The PCS was developed in 1995. The scale includes 13 items divided into three dimensions: rumination, magnification, and helplessness. Participants responded on a Likert scale ranging from 0 (not at all) to 4 (all times). Total PCS scores ranged from 0 to 52, with higher scores indicating more severe catastrophic thinking. Previous studies reported Cronbach’s alpha coefficients of 0.89 to 0.95 for the Chinese version of this scale [27].

### Extraction and purification of NPs from mouse tissue samples

Ultrapure water was used during the experiments, and all equipment and containers were thoroughly washed several times with particle-free water prior to use. The blank group for the mouse tissue experiment consisted of mice that received no treatment, whereas the experimental group included mice that were injected with F-PS NPs at a dosage of 50 mg/mL ten consecutive times [28]. Subsequently, mouse brain and spinal cord tissues were dissected. For the blank group, after adding the F-PS NPs standard, the procedure was the same as that used for the experimental group. Subsequently, 150 mg of brain tissue and 20 mg of spinal cord tissue were weighed and grounded, and 750 µL and 100 µL of water were added, respectively. Tissue samples were subjected to enzymatic hydrolysis and chemical digestion to remove the biological components. First, the tissue samples were mixed with trypsin (0.25%, w/v) at a ratio of 5:1 at 37 °C for 2 h. Subsequently, lipase (10 mg/mL) was added at 37 °C, and the enzymatic hydrolysis was conducted at a ratio of 5:1 for 6 h. Next, proteinase K (10 mg/mL) was added at 55 °C, and the enzymatic hydrolysis was performed at a ratio of 25:1 for 12 h. The tissue samples were mixed with particle-free Benzonase (2 KU/mL) at a ratio of 25:1 at 37 °C for 2 h to degrade nucleic acids. The resulting solution was centrifuged at 14,900 rpm for 30 min. The tissue precipitate was subjected to another round of enzymatic hydrolysis using trypsin, lipase, proteinase K, and Benzonase to ensure complete enzymatic hydrolysis. Finally, the enzyme-containing tissue samples were digested with TMAH (25%, v/v) at a 1:1 ratio for 12 h at room temperature. F-PS NPs were obtained by centrifuging the resultant solution at 14,900 rpm for 30 min, followed by a single wash with deionized water to remove residual impurities. Finally, the F-PS NPs were dispersed in 50 µL of ultrapure water and stored at 4 °C.

### Raman detection of F-PS NPs

The obtained F-PS NPs in 50 µL of solution were sonicated for 25 min to suspend the particles. Then, 5 µL of the analyte solution was pipetted onto a glass slide and dried at room temperature. Subsequently, a 785 nm laser beam with 10% laser power was directly focused on the coffee ring. Twenty Raman spectra were recorded for each group of samples. Asymmetric least-squares smoothing was utilized for spectral preprocessing to perform baseline correction, and the Savitzky–Golay smoothing algorithm with a window size of 5 was used for smoothing.

### Cell culture

The BV2 cell line was maintained in Roswell Park Memorial Institute (RPMI) 1640 medium (Gibco, Billings, MT, USA) supplemented with 10% fetal bovine serum (FBS) (ST30-3302; PAN-Biotech GmbH, Aidenbach Germany) and 1% penicillin/streptomycin (Beyotime Biotechnology). Cells were cultured in a humidified incubator at 37 °C with 5% CO₂.

### Behavior test

Von Frey test: Mice were acclimated to the Von Frey testing environment for at least 2 days prior to baseline testing. For testing, the animals were individually placed in transparent plastic boxes positioned on an elevated mesh von Frey apparatus and allowed to acclimate for at least 30 min before the procedure. Mechanical hypersensitivity was assessed using a series of von Frey filaments (UGO Basile, Gemonio, Italy) applied perpendicularly to the mid-plantar surface of the hind paw, with sufficient force to bend the filament into an S-shape, following the methodology established in our previous work. The “Up-Down” testing paradigm was used to determine the PWT. The difference between the baseline and test values was calculated using the analytical approach described in a previous study [29].

Cold- and hot-plate experiments: Environmental acclimation prior to testing was performed under the same conditions as in the von Frey test. During the procedure, the animals were individually placed on a hot plate and allowed to acclimate for at least 30 min before measurements. The paw withdrawal latency was recorded as the time elapsed until a positive nociceptive response was observed, following the methodology described in our previous study. A positive nociceptive response was defined as licking of the hind paw, sudden rapid movement, hind paw flinching, or immediate lifting of the paw [29].

Cotton swab, brush, and pinprick tests: Environmental adaptation before the experiment was identical to that of the von Frey test. The animals were individually placed in transparent plastic chambers on an elevated mesh von Frey frame and allowed to acclimate for at least 30 min before testing. Using brushes, cotton swabs, and pins, slow stimuli were applied to the center of the mouse hind paws as previously described. Positive nociceptive responses, defined as rapid paw lifting, hind paw licking, or quick movement, were recorded. A minimum interval of 5 min was maintained between consecutive tests [29]. A strict double-blind protocol was adopted for all behavioral tests, with coded grouping, dual-experimenter operation, and post-test data decoding.

### Assessment of Depressive-like behaviors in mice

All mice were acclimatized to the testing room for 30 min daily over one week prior to experiments. Behavioral tests were recorded and analyzed using Panlab SMART Video Tracking Software (v3.0).In the Open Field Test (OFT), mice explored a 50 × 50 × 40 cm arena (ethanol-sanitized between trials). We recorded 5-minute sessions starting from the center, measuring total distance traveled (locomotor activity) and time in the central zone (anxiety/depression-like behavior).The Elevated Plus Maze (EPM) featured two open arms (50 × 10 cm) and two enclosed arms (50 × 10 × 30 cm), similarly sanitized. Mice were placed at the junction, and 5-minute sessions assessed anxiety/depression-like behavior via time in open arms and open-arm entries.For the Tail Suspension Test (TST), mice were suspended by the tail (unable to touch surfaces). Immobility time during 5-minute sessions quantified depressive-like behavior across groups [29].

### Spinal cord slice Preparation and electrophysiological recordings

Spinal cord slice preparation and electrophysiological recordings were performed as previously described [29, 30]. Mice were anesthetized with 4% isoflurane, and lumbar spinal cords were rapidly dissected and placed into an ice-cold cutting solution containing (in mM) 234 sucrose, 3 KCl, 1.2 NaH₂PO₄, 11 glucose, 26 NaHCO₃, 1.2 MgCl₂•6 H₂O, and 2.5 CaCl₂•2 H₂O before being cut into 350 μm-thick slices. The slices were then incubated in artificial cerebrospinal fluid (aCSF) oxygenated with 95% O₂ and 5% CO₂ at 35 °C for 30 min, where the aCSF comprised (in mM) 126 NaCl, 3 KCl, 1.2 NaH₂PO₄, 11 glucose, 26 NaHCO₃, 1.2 MgCl₂·6 H₂O, and 2.5 CaCl₂·2 H₂O, and outer lamina II neurons were selected for recording. Excitatory postsynaptic currents (EPSCs) were recorded using whole-cell voltage-clamp techniques with an intracellular pipette solution containing (in mM) 140 potassium gluconate, 20 KCl, 2 MgCl₂·6 H₂O, 10 HEPES, 2 Na₂ATP, and 0.2 EGTA; spontaneous EPSCs (sEPSCs) were recorded at a holding potential of − 70 mV, and miniature EPSCs (mEPSCs) were recorded after sEPSCs stabilized in the presence of 10 µM bicuculline, 1 µM tetrodotoxin, and 50 µM AP5. All signals were acquired using a MultiClamp700B amplifier (Axon Instruments Inc., Union City, CA, USA). All drugs were prepared in aCSF and purchased from Hello Bio Inc. (Princeton, NJ, USA) [29].

### Immunofluorescence

After the behavioral tests, the mice were anesthetized by intraperitoneal injection of 1% sodium pentobarbital (euthanyl). On the surgical platform, mice were perfused transcardially with 20 mL saline, followed by 20 mL pre-cooled 4% paraformaldehyde (PFA). After perfusion, spinal cords were flushed from the spinal cavity with saline and fixed overnight in excess PFA at 4 °C. Tissues were then gradient-dehydrated at 4 °C in sterilized sucrose solutions (20% and 30% sucrose in PBS buffer) until they sank to the bottom, indicating successful dehydration. The tissues were embedded in the OCT compound and rapidly frozen in liquid nitrogen. Using a cryostat (Leica #CM1950), tissues were sectioned at 13 μm thickness and mounted onto adhesive slides. The prepared slides were baked at 60 °C for 1 h. After sectioning, slides were blocked for 1 h with PBS containing 10% goat serum, 3% BSA, and 0.5% Triton X-100. Primary antibodies were applied and incubated overnight, followed by fluorescent secondary antibody labeling. The slides were washed three times with PBST (PBS with 0.5% Triton X-100) for 10 min each before and after each antibody incubation. Finally, the slides were mounted with a DAPI-containing mounting medium (Beyotime Biotechnology), and fluorescence imaging was performed using a Zeiss high-resolution confocal microscope [30]. For statistical analysis, the spinal cord region examined was laminae Ⅰ to Ⅳ of the ipsilateral dorsal horn in each animal. All IBA1-immunopositive cells (including DAPI-positive cells) were counted. Similarly, all neurons displaying clear nuclear c-FOS immunopositively signals were counted. Immunofluorescence data were analyzed double – blindly by two analysts with coded images, unified criteria and consistency verification.

### Western blot analysis

After all the behavioral tests were completed, the mice were anesthetized with an overdose of isoflurane and rapidly decapitated. The spinal cord and DRG (L3-L5) were dissected. Tissues were homogenized in RIPA buffer containing protease and phosphatase inhibitors (1% PMSF, Beyotime Biotechnology). The homogenate was centrifuged at 12,000 × *g* at 4 °C, and the supernatant was collected. Protein concentration was measured by BCA assay (Beyotime Biotechnology), with at least 40 µg of protein loaded per lane. Proteins were separated using 10% SDS-PAGE and transferred to PVDF membranes with 0.22–0.45 μm pore sizes. Blots were blocked with 5% non-fat milk at room temperature for 1 h, followed by overnight incubation with primary antibodies at 4 °C. After washing thrice with TBST, the blots were incubated with horseradish peroxidase-conjugated secondary antibodies at room temperature for 1 h and washed thrice with TBST. Protein expression levels were normalized to that of GAPDH and analyzed using ImageJ software (USA) [30].

### Real-time quantitative PCR

Spinal cords (L3-L5) were collected, quick-frozen in liquid nitrogen, and stored at − 80 °C. Total RNA was extracted using the TRIzol reagent (Thermo Fisher Scientific, Waltham, MA, USA). Total RNA was reverse transcribed using a reverse transcription kit (#R222–01, Vazyme, Nanjing, China). qPCR was conducted using a CFX96 real-time system (Bio-Rad Laboratories, Hercules, CA, USA) with the Universal SYBR qPCR master mix (#Q712–02, Vazyme). Each reaction was performed in triplicate and normalized to β-actin gene expression. Relative expression was determined using the ΔΔCT method [30].

### RNA sequencing

After the experimental procedures, the spinal cords from male mice were collected and snap-frozen. Individual mice were used as biological replicates (3 mice per genotype). The RNA quantity and integrity were assessed using the RNA Nano 6000 Assay Kit on a Bioanalyzer 2100 system (Agilent Technologies, Santa Clara, CA, USA). Library fragments were purified using an AMPure XP system (Beckman Coulter, Brea, CA, USA). mRNA was purified from the total RNA using poly T oligo-attached magnetic beads. Library fragments were purified using the AMPure XP system (Beckman Coulter). Following PCR amplification, the products were purified using AMPure XP beads to obtain a final library. The library was quantified using a Qubit 2.0 Fluorometer and sequenced on an Illumina NovaSeq 6000. The image data from the high-throughput sequencer were converted into sequence reads using CASAVA base calling. The reference genome was indexed using Hisat2 (v2.0.5), and reads mapped to each gene were counted using featureCounts (v1.5.0-p3). Statistical enrichment of differentially expressed genes in KEGG and GO pathways was analyzed using the clusterProfiler R package (v3.8.1) and ranked by the number of enriched genes [30].

### Pull-down and CO-IP

The PS NPs pull-down was performed in a manner similar to that used for Co-IP. Prewashed PS NPs (1 mg in 0.5% Tween-20 in PBS) was added to 0.5 mL the spinal cord protein extract (NP40, Beyotime Biotechnology). Control antibodies against P38, ERK, and JNK (1:50) were incubated with magnetic beads (Thermo Fisher Scientific, USA) at room temperature for 1 h. The beads were then added to 0.5 mL of spinal cord protein extract. For the control group, rabbit IgG (1:50, Beyotime Biotechnology) was added and incubated overnight at 4 °C. After incubation, samples were washed five times with PBST for 2 min each, followed by centrifugation at 12,000 × *g* for 15 min at 4 °C (PS NPs pull-down samples were centrifuged after each wash). Proteins were eluted by heating at 99 °C for 10 min in loading buffer containing β-mercaptoethanol [31].

### Molecular dynamics simulations

Molecular dynamics (MD) simulations were performed using GROMACS (version 2021.3) with the AMBER99SB force field. The PS NPs were composed of 1600 polystyrene molecules. Specifically, 80 polystyrene chains were evenly distributed in a 10-nm sphere using Packmol [32]. Heating and annealing were then performed using GROMACS to obtain the equilibrium structure of the PS NPs. ROSETA2020 was used for docking followed by MD simulations [33]. The protein and PS NPs were solvated in cubic water boxes containing 0.15 M NaCl to approximate physiological ionic conditions. System energy minimization was conducted in two stages: 5,000 steps using the steepest descent algorithm, followed by 5,000 steps of conjugate gradient optimization to resolve steric clashes. Equilibration was achieved through sequential relaxation under canonical, constant volume (NVT), isothermal-isobaric, 1 atm, and 300 K (NPT) ensembles, each for 2 ns, with periodic boundary conditions applied throughout. A 100-nanosecond production run was carried out with a 2-femtosecond time step, and trajectory snapshots were saved every 10 ps for downstream analysis [34].

### Data statistics

Statistical analyses were performed using GraphPad Prism 8.0. A two-tailed Student’s t-test was used for comparison between the two groups. Two-way ANOVA or multiple comparison tests with Bonferroni post-hoc tests were applied for three or more conditions. Statistical significance was set at *P* < 0.05.

## Results

### Workers in plastic manufacturing facilities exhibit heightened pain sensitivity

**Fig. 1:**
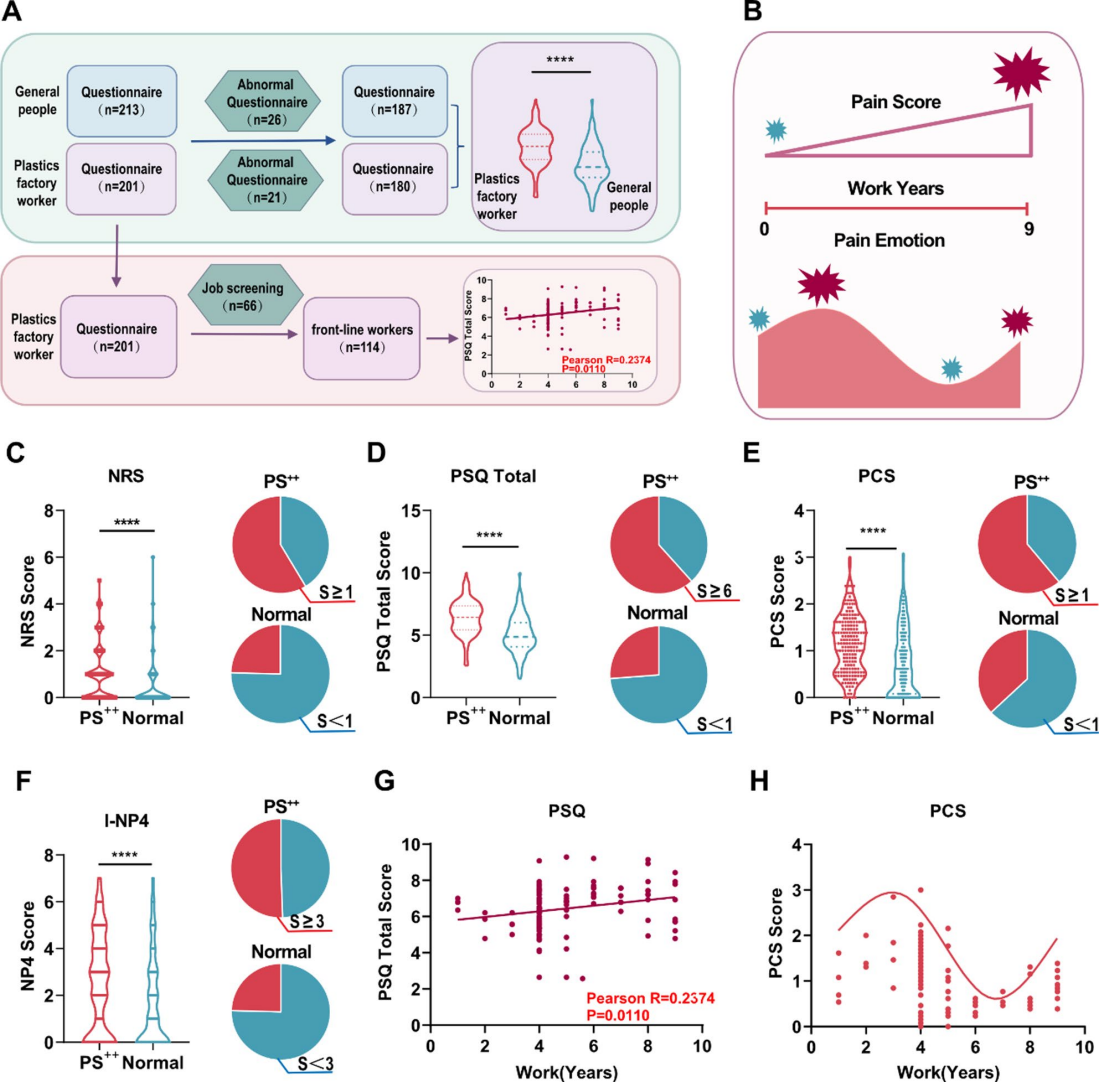
Workers in plastic manufacturing facilities exhibit heightened pain sensitivity (**A**) Flowchart of questionnaire screening procedures; (**B**) Representative plots of correlations between PSQ/PCS scores and duration of frontline work experience; (**C**) Numerical Rating Scale (NRS) scores (*n* > 180); (**D**) Pain Sensitivity Questionnaire (PSQ) scores; (**E**) Pain Catastrophizing Scale (PCS) scores; (**F**) Interventional Neuropathic Pain 4-Item (I-NP4) scores; (**G**) Correlation analysis between PSQ scores and duration of frontline work experience; (**H**) Correlation analysis between PCS scores and duration of frontline work experience; Data are presented as mean ± SEM. ****p < 0.001

Pain assessment scales are validated and reliable tools for evaluating pain characteristics and progression, which have been widely utilized in clinical trials, diverse hospital settings, and specialized pain clinics. Specifically, the Numeric Rating Scale (NRS) quantifies pain severity [25], while the I-NP4 identifies neuropathic pain components [26]. To investigate whether there is an association between workers in plastic manufacturing facilities and pain, we recruited workers from plastic product manufacturing plants and collected 201 questionnaires, which included NRS, Perceived Stress Questionnaire (PSQ) [35], Pain Catastrophizing Scale (PCS) [27], and I-NP4. After excluding 21 questionnaires based on chronic disease criteria and aberrant responses, 180 occupationally exposed individuals were included. In the general population, 213 questionnaires were obtained through non-selective recruitment, of which 26 were excluded after screening, resulting in 187 controls (Fig. 1A, B).

NRS questionnaire data showed that the mean NRS score was 0.97 ± 0.085 in the occupationally exposed population, significantly higher than 0.40 ± 0.066 in the general population. An NRS score ≥ 1 was defined as persistent pain in the past month, and 57% of occupationally exposed individuals reported prolonged pain versus only 25% of the general population (Fig. 1C). Additionally, PSQ scale results indicated that the mean PSQ score was 6.40 ± 0.101 in the exposed group, higher than 5.08 ± 0.110 in controls. A PSQ score ≥ 6 was classified as paresthesia, with 62% of the occupationally exposed population exhibiting this symptom compared to 26% of controls (Fig. 1D). Concurrently, PCS scale data revealed that the mean PCS score was 1.14 ± 0.046 in exposed workers, significantly higher than 0.78 ± 0.052 in the control group. A PCS score > 1 was defined as excessive negative emotions toward pain, and 61% of the occupationally exposed individuals demonstrated such responses compared to 37% of the controls (Fig. 1E). Finally, I-NP4 scale results showed that the mean score was 2.50 ± 0.156 in the exposed population, significantly higher than 1.66 ± 0.141 in the general population. An I-PN4 score ≥ 3 was suggestive of possible neuropathic pain, with 50% of occupationally exposed individuals meeting this criterion compared to 25% of controls (Fig. 1F).

From the occupationally exposed cohort, 114 questionnaires completed by the frontline production workers were selected for further analysis. A significant positive correlation was observed between the years of employment and PSQ scores (*p* = 0.011), indicating that sensory abnormalities increased with prolonged occupational exposure (Fig. 1G). Analysis of PCS scores revealed a biphasic relationship with employment duration; initial low-level negative emotions toward pain gradually intensified as pain prevalence increased. However, subsequent adaptation to chronic pain temporarily reduced the PCS scores, followed by a resurgence of maladaptive emotional responses as pain severity progressed (Fig. 1H). Collectively, our findings demonstrated that workers in plastic-related industries exhibit heightened sensory abnormalities, dysregulated emotional responses to pain, and an elevated likelihood of neuropathic pain compared with the general population.

### PS NPs induce hyperalgesia in mice

We prepared PS NPs by a reported polymerization reaction method [36]. For comparation, fluorescent labeling PS (F-PS) NPs were prepared by swelling with Nile red. The size (~ 50 nm), surface charge (PS NPs, − 21.7 mV; F-PS NPs, -19.8 mV), FT-IR spectra, Raman spectra, fluorescence spectra, and stability of these NPs are presented in Sect "Workers in plastic manufacturing facilities exhibit heightened pain sensitivity" in Supporting information (SI).

**Fig. 2:**
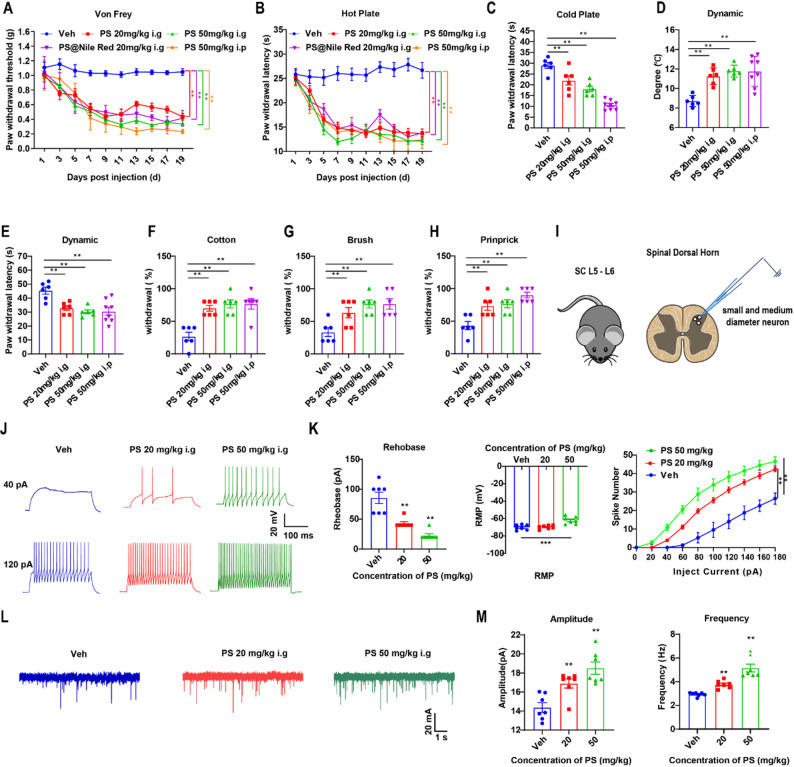
PS NPs-induced mechanical and thermal hypersensitivity in mice via intraperitoneal injection and oral gavage. (**A)** Time-dependent changes in mechanical paw withdrawal threshold; (**B**) Time-dependent changes in thermal paw withdrawal latency; (**C**) Cold paw withdrawal latency in PS NPs -treated mice; (**D-E**) Dynamic temperature test results of cold plate (temperature vs. time threshold); (**F**) Cotton swab test; (**G**) Brush test; (**H**) Pinprick test (*n* > 6) ; (**I**) Schematic of electrophysiological recordings from small-diameter neurons in the dorsal horn of L5-L6 spinal cord; (**J**) Representative action potential traces of dorsal horn neurons under 20 pA and 120 pA current injections; (**K**) Rheobase, resting membrane potential, and action potential changes with current injection in dorsal horn neurons (*n* = 7); (**L**) Representative traces of spontaneous excitatory postsynaptic currents (sEPSC); (**M**) Amplitude and frequency of sEPSC in dorsal horn neurons (*n* = 7). Data are expressed as mean ± SEM. *p < 0.05, **p < 0.01 vs. WT group

In order to investigate the biodistribution of PS NPs in various murine organs, in vivo imaging was conducted by using F-PS NPs. In vivo imaging revealed systemic distribution of F-PS NPs across superficial regions of mouse body surfaces. Subsequent fluorescence imaging of dissected organs demonstrated F-PS NPs accumulation in the brain, spinal cord, liver, spleen, kidneys, lungs, testes, and serum, whereas no detectable fluorescence was observed in cardiac tissues (Figure S5). Further, in order to quantitative detect the PS NPs in these tissues, we first established a Raman-based detection method via continuous enzymatic tissue digestion. The quantitative results revealed that the PS NPs content in the spinal cord of mice was higher than that in the brain (Sect. "PS NPs induce hyperalgesia in mice" and "PS NPs alter the inflammatory milieu in the mouse spinal dorsal Horn" in SI). Due to the superior bioavailability of intraperitoneal (i.p.) injection compared with intragastric administration—primarily because a significant portion of orally delivered PS NPs is excreted unabsorbed in feces—we established a pilot model in which PS NPs were administered via i.p. injection at varying concentrations (0–50 mg/kg) over 15 days to observe their effects on nociceptive behavior (Figure S6A). Both the mechanical paw withdrawal threshold (PWT) and thermal paw withdrawal latency (PWL) showed a dose-dependent reduction (Figure S4B-D). Consequently, we selected 20 and 50 mg/kg PS NPs for subsequent experiments [37] while validating the functional equivalence between fluorescently labeled F-PS NPs (20 mg/kg) and PS NPs. Behavioral analyses revealed no significant differences in the mechanical or thermal sensation thresholds between the intragastric (i.g.) (50 mg/kg) and i.p. (50 mg/kg) PS NPs groups (Fig. 2A, B), with comparable thresholds observed between the F-PS NPs (20 mg/kg, i.g.) and PS NPs (20 mg/kg, i.g.) groups (Fig. 2A, B). We used cold plate testing, cotton swab assay, dynamic hyperalgesia (brush stimulation), and pinprick hyperalgesia to confirm PS-induced sensory abnormalities further. PS NPs-treated mice (i.g. or i.p.) exhibited heightened sensitivity to both cold and noxious stimuli (Fig. 2C-F). In contrast, PS NPs pretreatment increased responsiveness to non-noxious stimuli (cotton swab and brush), manifested as elevated mechanical response frequency and reduced cold response thresholds (Fig. 2G, H). We also examined the effects of PS NPs in mice of different sexes (i.n.). The results showed that inhaled PS NPs induce pain hypersensitivity in a sex-independent manner (Figure S7). Given that altered neuronal excitability and synaptic transmission are hallmarks of pain pathophysiology, we performed spinal cord patch-clamp recordings of small-diameter dorsal horn neurons following PS NPs intervention (Fig. 2I). Under voltage-clamp mode with stepwise current injections (− 60 to 200 pA, 20 pA increments, 5 s intervals), rheobase values significantly decreased in both 20 mg/kg and 50 mg/kg PS NPs groups (mean < 60 pA required to elicit action potentials), resting membrane potentials showed marked depolarization in the 50 mg/kg PS NPs group, and action potential firing frequency increased significantly across 0-200 pA stimulations (Fig. 2J, K). Additionally, PS NPs treatment elevated both the frequency and amplitude of spontaneous excitatory postsynaptic currents (sEPSCs) (Fig. 2L, M), indicative of enhanced synaptic transmission.

### PS NPs alter the inflammatory milieu in the mouse spinal dorsal Horn

**Fig. 3:**
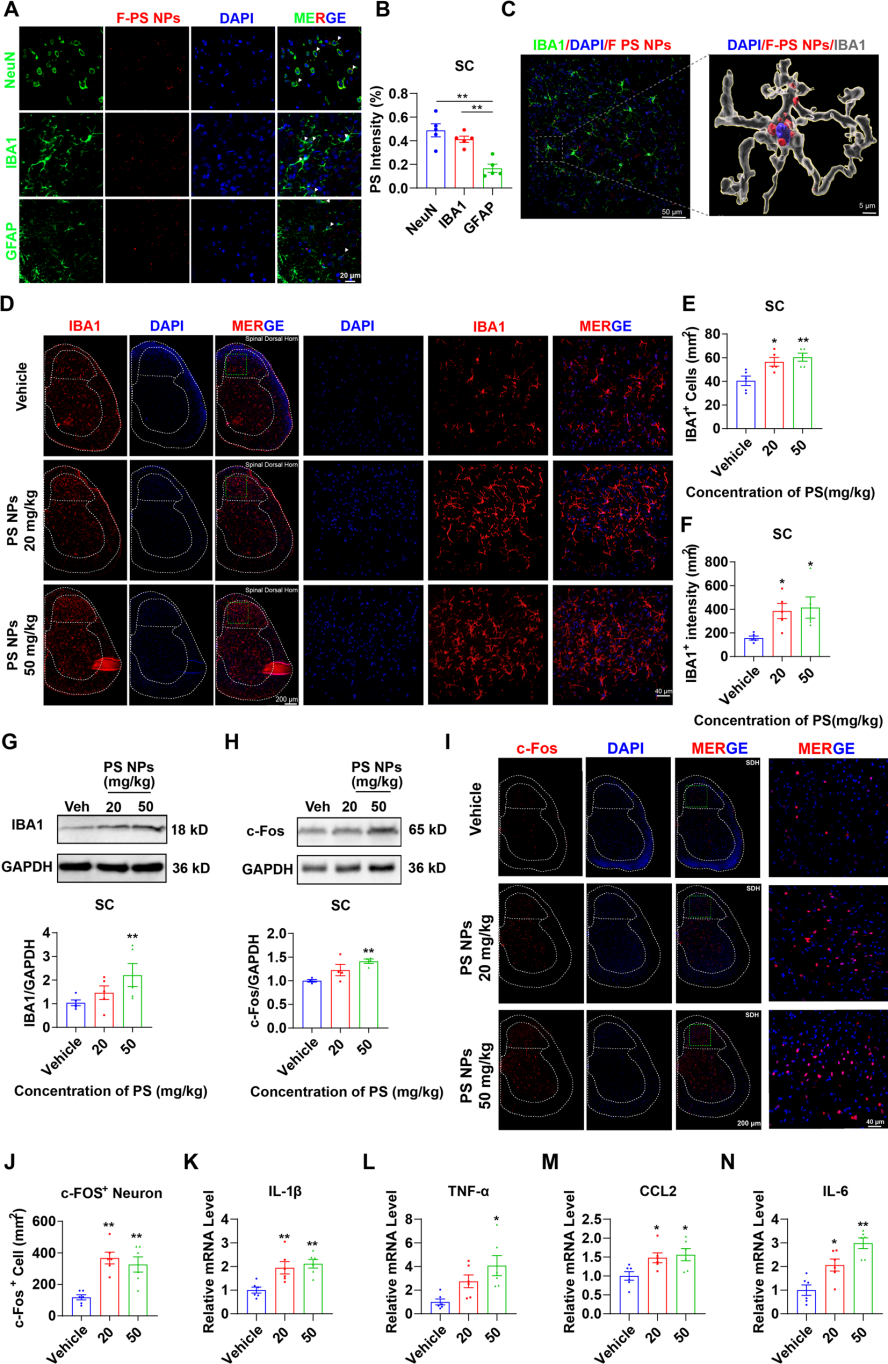
PS NPs increased microglial reactivity in the mouse spinal cord. (**A**) Immunofluorescence staining of mouse spinal cord tissues showing individual and merged channels for the neuronal marker NeuN, microglial marker IBA1, astrocytic marker GFAP, F-PS NPs, and the nuclear dye DAPI; (**B**) Distribution of F-PS NPs within spinal cord neurons, microglia, and astrocytes (n=5); (**C**) Immunofluorescence colocalization images of IBA1, DAPI, and F-PS NPs (left panel) with a magnified view (right panel), clearly showing the intracellular distribution of F-PS NPs within microglia; (**D**) Representative immunofluorescence images of microglia (IBA1 staining) in the spinal dorsal horn from Vehicle, 20 mg/kg PS NPs, and 50 mg/kg PS NPs groups.(**E**) Quantitative analysis of microglial numbers in the spinal dorsal horn (n=5); (**F**) Quantitative analysis of IBA1 fluorescence intensity in the spinal dorsal horn (n=5); (**G**) Western blot analysis of IBA1 protein levels in the spinal cord across treatment groups (normalized to GAPDH), with corresponding quantitative results of relative protein expression (n=5); (**H**) Western blot analysis of c-FOS protein levels in the spinal cord across treatment groups (normalized to GAPDH), with corresponding quantitative results of relative protein expression (n=5); (**I**) Representative immunofluorescence images of c-FOS-positive neurons in the mouse spinal cord from Vehicle, 20 mg/kg PS NPs, and 50 mg/kg PS NPs groups; (**J)** Quantitative analysis of c-FOS-positive neuron numbers in the spinal dorsal horn (n=5); (**K-N)** Quantitative mRNA expression levels of the inflammatory cytokines IL-1β (**K**), TNF-α (**L**), CCL2 (**M**), and IL-6 (**N**) in the spinal dorsal horn (n=5). All data are presented as mean ± SEM; *p < 0.05, **p < 0.01 vs. the Vehicle group

Following the administration of F-PS NPs (i.g.), mice were perfused, and spinal cord tissues were collected for cryosectioning to analyze the distribution of the nanoparticles. F-PS NPs showed co-localization with the neuronal marker NeuN, the microglial marker IBA1, and the astrocytic marker GFAP. Quantitative analysis revealed that approximately 48% of the internalized F-PS NPs were located within neurons, 42% within microglia, and 16% within astrocytes (Fig. 3A, B). To further confirm the internalization of F-PS NPs by microglia, 3D reconstruction was performed, which verified the presence of nanoparticles within the microglial cell bodies (Fig. 3C).

Subsequently, to further investigate the impact of PS NPs on spinal microglia, we performed immunofluorescence staining and Western blot analysis on spinal cord tissues from PS NP-treated mice. The results demonstrated that PS NPs significantly increased microglial reactivity in the spinal dorsal horn. This was evidenced by increased IBA1^+^ cell counts, enhanced IBA1 fluorescence intensity (Fig. 3D-F) and elevated IBA1 protein expression (Fig. 3G).

Considering that neuronal hyperactivation is a hallmark of pain states, we further evaluated the activation of spinal dorsal horn neurons under PS NPs intervention using c-Fos as a neuronal activity marker. Immunofluorescence and western blot analyses consistently showed that PS NPs treatment robustly enhanced neuronal activation, which was characterized by an increased number of c-Fos + neurons and elevated c-Fos protein expression (Fig. 3H-J).

Given the established association between heightened microglial reactivity and neuroinflammation, we examined the expression of inflammatory mediators in the DH of the spine. qRT-PCR analysis revealed that PS NPs treatment markedly upregulated the mRNA levels of IL-1β, IL-6, TNF-α, and CCL2 (Fig. 3K-N), To further determine the contribution of altered spinal inflammatory homeostasis to PS NP-induced pain hypersensitivity, we performed intrathecal injection of 3 µg PS NPs into the L4-L6 intervertebral spaces in mice (i.t.). Tissues, including the lumbar spinal cord, thoracic spinal cord, lumbar dorsal root ganglia, sciatic nerve, medulla oblongata, cerebral cortex, and serum, were collected 24 h post-injection. The protein levels of iNOS and IL-1β in these tissues were measured by ELISA. The results showed that intrathecal PS NPs rapidly induced pain hypersensitivity in mice (1 day). Notably, inflammatory changes were observed specifically in the spinal cord one day after injection. This confirms that PS NP-induced disruption of spinal inflammatory homeostasis is a core driver of pain hypersensitivity in mice (Figure S8).

### Microglial depletion rescues PS NPs-induced hyperalgesia in mice

**Fig. 4:**
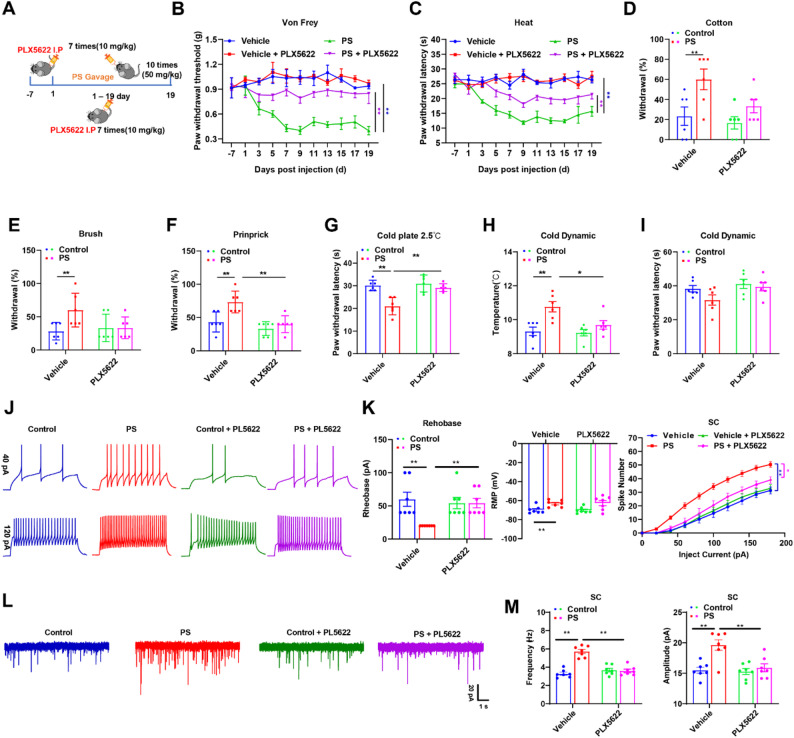
Microglia depletion rescued PS NPs -induced hyperalgesia in mice. (**A**) PLX5622 and PS NPs administration protocols; (**B**) Time-dependent changes in mechanical paw withdrawal thresholds and (**C**) thermal paw withdrawal latencies. Post-treatment behavioral assessments included cotton swab test (**D**), brush test (**E**), pinprick test (**F**), cold withdrawal latency (**G**), and dynamic temperature test (temperature vs. time threshold) (**H,I**) (n= 6);(**J**) Representative action potential traces of spinal dorsal horn neurons under 20 pA and 120 pA stimulation; (**K**) Rheobase, resting membrane potential, and action potential firing patterns of spinal dorsal horn neurons with increasing current injections (n= 7);(**L**) Representative traces of spontaneous excitatory postsynaptic currents (sEPSCs); (**M**) Amplitude and frequency of sEPSCs in spinal dorsal horn neurons (n= 7). Data are expressed as mean ± SEM. *p < 0.05, **p < 0.01

**Fig. 5:**
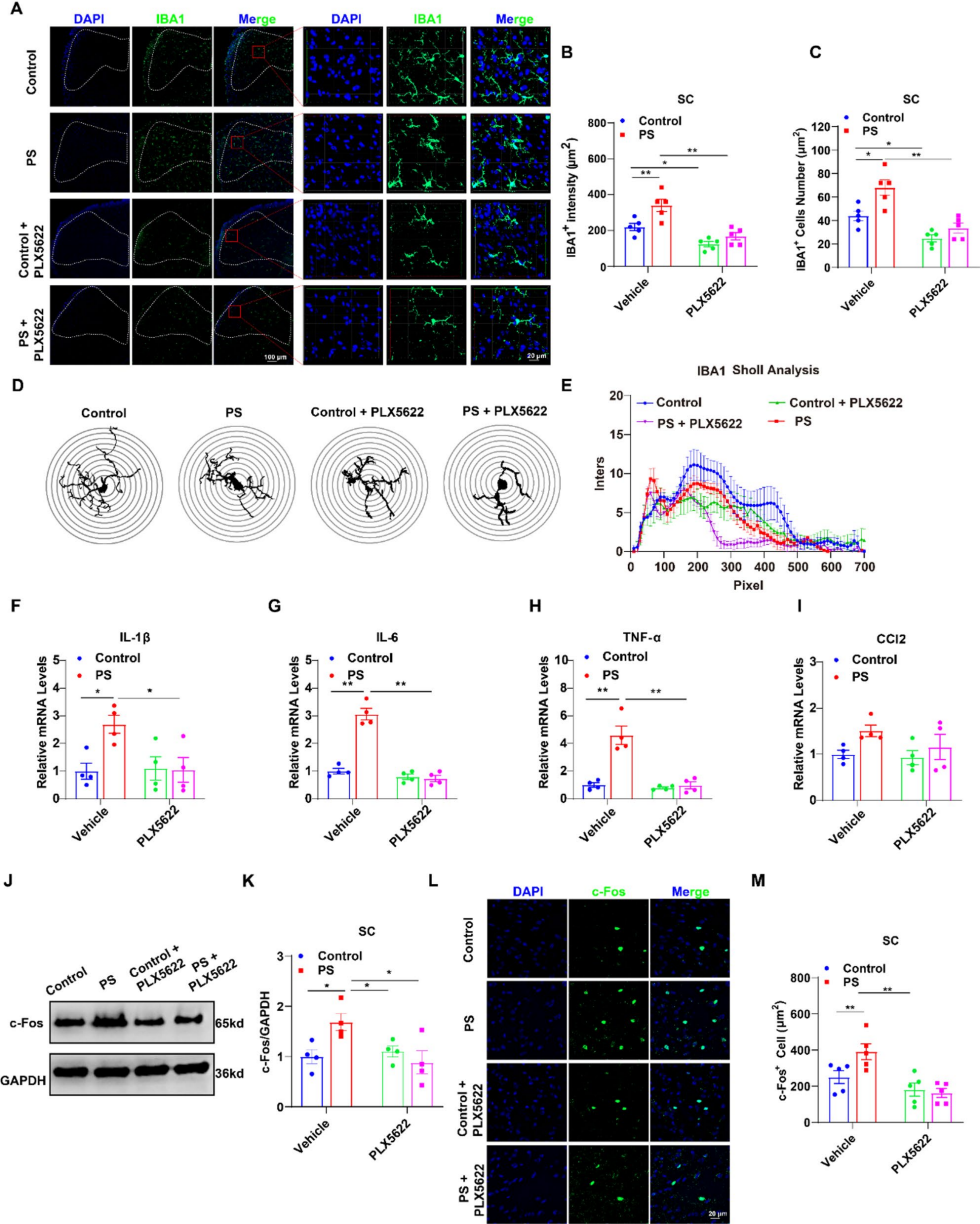
Microglia depletion attenuated PS NPs-induced inflammatory activation in the mouse spinal dorsal horn. (**A**) PLX5622 treatment reduced the number of microglia (**B**) and decreased IBA1 fluorescence intensity (**C**) in the spinal dorsal horn (n= 5); (**D,E**) Sholl analysis demonstrated that PS NPs -treated microglia exhibited reduced synaptic complexity compared to controls, whereas PLX5622-treated microglia displayed dystrophic features, including smaller somata and similarly diminished synaptic complexity (n= 5). Following PLX5622 administration, mRNA levels of inflammatory cytokines in the spinal dorsal horn were altered: IL-1β (**F**), IL-6 (**G**), TNF-α (**H**), and CCL2 (**I**) (n= 4); (**J,K**) Changes in c-FOS protein expression in the spinal dorsal horn (n= 4);(**L**) Representative immunofluorescence images of c-FOS-positive cells in the spinal cord; (**M**) Quantification of c-FOS-positive neurons in the spinal dorsal horn (n> 5). Data are expressed as mean ± SEM. *p < 0.05, **p < 0.01

Microglial depletion is a viable therapeutic approach for neuroinflammation-related diseases [38], Thus we performed pharmacological microglial ablation to investigate whether microglial reactivity contributed to PS NPs-induced hyperalgesia. PLX5622, a CSF1R antagonist, was intraperitoneally administered 7 days prior to PS NPs injection, and subsequently, every other day post-PS NPs injection until behavioral assessments were completed (Fig. 4A). This regimen achieved approximately 50% microglial depletion in spinal cord tissue (Fig. 5A-C). Analysis of microglial morphology via Sholl analysis indicated that PS NPs heightened microglial reactivity, evidenced by hypertrophy and simplified arborization. Crucially, depleting microglia with PLX5622 did not prevent PS NPs from inducing this reactive phenotype in the remaining cells. This supports the conclusion that the quantity of microglia, not merely their activation state, is the key determinant in modulating PS NPs-induced pain hypersensitivity (Fig. 5D-E). Notably, microglial inhibition coincided with downregulated expression of spinal inflammatory mediators (IL-1β, IL-6, TNF-α, and CCL2) in PS NPs-treated mice (Fig. 5F-I), accompanied by reduced neuronal activation in the spinal dorsal horn (Fig. 5J-M).

Notably, PLX5622-treated and control male mice exhibited comparable baseline mechanical and thermal sensitivity, indicating microglial depletion did not affect basal nociception. However, PLX5622 administration markedly alleviated PS NPs -induced mechanical hyperalgesia and thermal hyperalgesia compared to controls (Fig. 4B-I). Electrophysiological recordings further demonstrated that microglial depletion significantly reduced the PS NPs-triggered hyperexcitability of spinal dorsal horn neurons and attenuated the frequency and amplitude of synaptic transmission (Fig. 4J-M). Collectively, these findings established that established that a shift in microglial state is a critical driver is a critical driver of PS NPs-induced hyperalgesia initiation and maintenance.

Critically, PS NPs-induced hyperalgesia emerged on post-injection day three, whereas depressive behaviors manifested on day 28. This temporal dissociation indicates that nociceptive abnormalities precede neuropsychiatric symptoms. Remarkably, Microglial depletion mitigated PS NPs-induced hyperalgesia and significantly ameliorated anxiety-like/depression-like behaviors in subsequent behavioral tests (Figure S9).

### PS NPs-induce activation of the MAPK pathway in the mouse spinal dorsal Horn

**Fig. 6:**
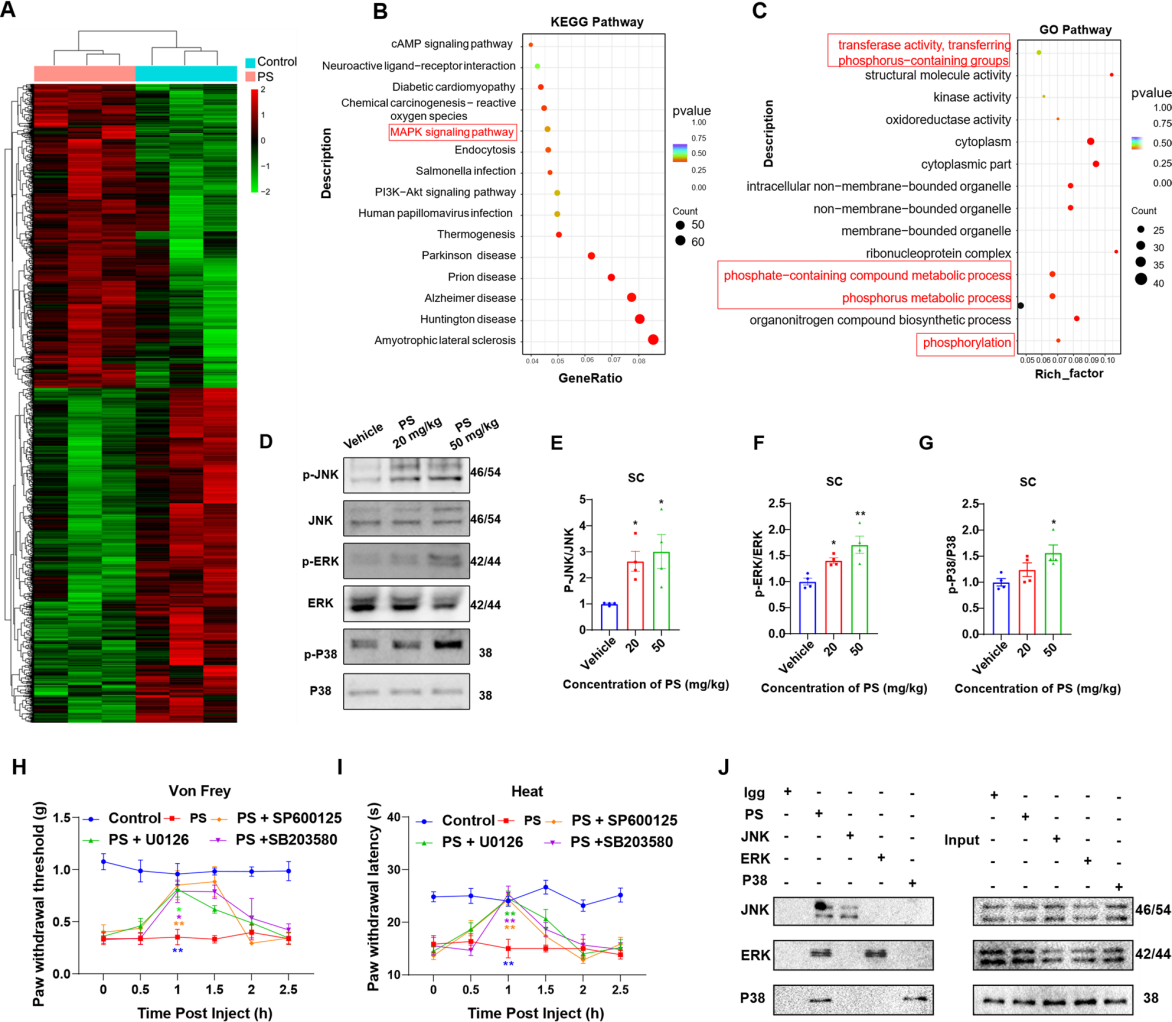
PS NPs induces activation of the MAPK pathway in the mouse spinal dorsal horn. (**A**) Heatmap of differentially expressed genes in the spinal dorsal horn of mice treated with 50 mg/kg PS NPs (RNA-seq,n= 3); (**B**) Kyoto Encyclopedia of Genes and Genomes (KEGG) Pathway enrichment analysis. (**C**)Gene Ontology (GO) functional enrichment analysis of differentially expressed genes; (**D**) Activation levels of the MAPK pathway in the spinal dorsal horn after PS NPs treatment (n= 4); (**E**) JNK, (**F**) ERK, and (**G**) P38 protein expression; (**H,I**) Effects of intrathecal injection of MAPK pathway inhibitors on mechanical paw withdrawal threshold and thermal paw withdrawal latency in mice within 2 h (n= 6); (**J**) Pull-down assay revealed direct interactions between PS NPs and JNK, ERK, and P38. Data are presented as mean ± SEM. *p < 0.05, **p < 0.01

To elucidate the molecular mechanisms underlying PS NPs-induced sensory abnormalities in mice, RNA sequencing was used to profile gene expression in the spinal dorsal horn of mice following PS NPs intervention to determine the mechanism by which PS NPs induces increased microglial reactivity (Fig. 6A). Kyoto Encyclopedia of Genes and Genomes (KEGG) pathway enrichment analysis revealed a significant increase in the expression of genes associated with the MAPK pathway (Fig. 6B). Gene Ontology (GO) functional annotation analysis revealed extensive alterations in signaling pathways related to phosphorylation processes (Fig. 6C). Western blot analysis confirmed that the phosphorylation levels of the MAPK pathway mediated by ERK, JNK, and p38 (three highly homologous kinases) in the spinal dorsal horn were significantly higher in PS NPs -treated mice than in controls (Fig. 6D–G). Notably, the pharmacological inhibition of MAPK components using SB203580 ( p38 inhibitor), U0126 ( MEK1/2 inhibitor), and SP600125 ( JNK inhibitor) significantly alleviated PS NPs -induced hyperalgesia (Fig. 6H, I). These results indicate that the activation of the MAPK pathway is a critical factor in PS NPs-induced hyperalgesia in mice. Pull-down assays confirmed the interactions between PS NPs and ERK, JNK, and p38 (Fig. 6J). Correspondingly, we investigated the effects of PS NPs on MAPK pathway activation and inflammatory responses in BV-2 cells. The results demonstrated that PS NP exposure significantly induced both inflammatory activation and MAPK pathway upregulation in this microglial model (Figure S10).

**Fig. 7:**
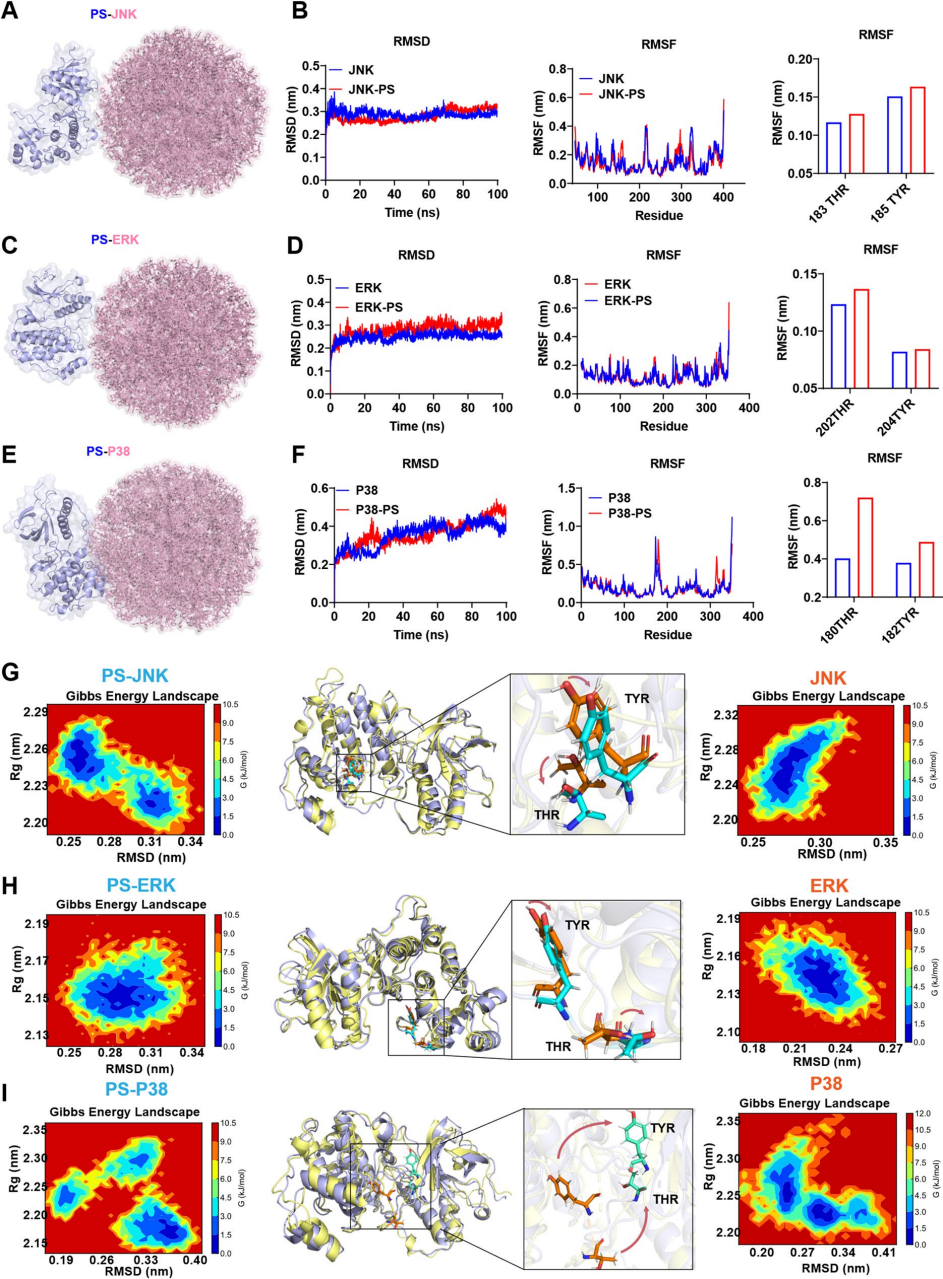
PS NPs enhances the flexibility of THR-X-TYR amino acid residues by binding to mitogen-activated protein kinase (MAPK) pathway proteins. (**A**) Schematic illustration of the binding between JNK (c-Jun N-terminal kinase) and PS NPs; (**B**) Analysis of root-mean-square deviation (RMSD) and root-mean-square fluctuation (RMSF) of JNK protein in the presence and absence of PS NPs, with a focus on the flexibility changes of its THR-X-TYR amino acid residues; (**C**) Schematic illustration of the binding between ERK (extracellular signal-regulated kinase) and PS NPs; (**D**) Analysis of RMSD and RMSF of ERK protein in the presence and absence of PS NPs, highlighting the flexibility alterations of THR-X-TYR amino acid residues; (**E**) Analysis of RMSD and RMSF of P38 protein in the presence and absence of PS NPs, with emphasis on the flexibility changes of its THR-X-TYR amino acid residues; (**F**) Schematic illustration of the binding between P38 and PS NPs; (**G-I**) Analysis of Gibbs free energy during the simulation to generate Gibbs free energy landscapes, extraction of lowest-energy conformations, and comparison of conformational changes in JNK, ERK, and P38 proteins in the presence and absence of PS NPs

Molecular docking and molecular dynamics simulations were performed to analyze the interactions between the PS NP sand ERK, JNK, and p38 and investigate how PS NPs regulates ERK, JNK, and p38 phosphorylation. The initial conformations obtained from simulations are shown in (Fig. 7A, C, E). The root-mean-square deviation (RMSD) was used to monitor conformational changes relative to the initial structure during the simulation, with the system reaching equilibrium after 10 ns of simulation Root-mean-square fluctuation (RMSF) reflects the flexibility of amino acid residues in proteins, with higher flexibility indicating a greater likelihood of being captured and phosphorylated by upstream kinases. Analysis of the flexibility of the THR-X-TYR amino acid motifs in JNK, ERK, and p38 proteins during the simulation showed that the presence of PS NPs enhanced the flexibility of these motifs (Fig. 7B, D, F).

Further analysis of the free energy changes during molecular dynamics simulations was used to construct Gibbs free energy landscapes and extract the lowest-energy conformations. In the presence of PS NPs, the hydroxyl groups in the THR-X-TYR motifs of the ERK, JNK, and p38 proteins exhibited flexible outward shifts from the protein interior. This may reduce the recognition and binding barriers for upstream kinases to phosphorylate these motifs (Fig. 7G-I). Taken together, PS NPs likely promotes the exposure of phosphorylation sites and enhances the phosphorylation of ERK, JNK, and xp38 by increasing the flexibility of their THR-X-TYR motifs.

## Discussion

The biological toxicity induced by polystyrene (PS) microplastics has emerged as a critical public health challenge in the 21st century. Recent studies have demonstrated a persistent increase in nanoplastic detection in human brain tissue from 2016 to 2024. Notably, the average microplastic concentration in human brains reached 4000 µg/g tissue by 2024, with Alzheimer’s disease patients showing significantly higher cerebral microplastic loads than healthy controls [9]. Importantly, these levels substantially exceed those observed in the central nervous systems of experimental mouse models (brain tissue: 168.4 µg/g; spinal cord: 354.2 µg/g; serum: 1000 µg/g), despite potential explanations such as the larger average particle size of accumulated microplastics in humans compared to standardized particles used in laboratory settings. More critically, if environmental microplastics remain unmitigated, their low metabolic clearance rates may drive bioaccumulation toward unprecedented thresholds over time.

In this study, we revealed for the first time that high-dose PS NPs exposure induces significant pain-related hypersensitivity (hyperalgesia) in mice. Notably, this effect may be mediated by direct activation of the MAPK pathway, leading to altered inflammatory conditions in the spinal cord. Furthermore, activation of the MAPK pathway is involved in the progression of virtually all pain models. PS NPs exposure also triggers spinal neuronal activation and excitability changes, indicating that PS NPs-induced central sensitization shares striking similarities with other pain models. Both patients and animal models exhibited pronounced sensory abnormalities following 1–2 administrations of chemotherapeutic agents, consistent with preclinical chemotherapy-induced neuropathic pain models and clinical observations. Importantly, these findings parallel our questionnaire-based results in populations exposed to occupational PS NPs.

Microglial depletion therapy has demonstrated potential therapeutic value across various preclinical disease models. For example, in mouse models of Alzheimer’s disease, this approach significantly alleviates pathological damage and improves cognitive function [39]. It also mitigates immune rejection in acute graft-versus-host disease (aGVHD) and ameliorates mechanical and thermal hypersensitivity in a mouse model of spinal nerve transection (SNT) [40]. In this study, we employed a pharmacological method to specifically ablate microglia to investigate their role in polystyrene nanoparticle (PS NP)-induced pain hypersensitivity. Our results show that microglial depletion significantly alleviated PS NP-induced pain behaviors. The mechanism primarily involves antagonizing the ATP-binding site of the Colony Stimulating Factor 1 Receptor (CSF1R) on microglia, which blocks downstream survival signals, inhibits microglial viability and self-renewal [41], and ultimately induces apoptosis. This process leads to a marked reduction in microglial numbers in the spinal cord, thereby improved local neuroinflammatory environment, and consequently, the alleviation of pain hypersensitivity [42].

However, it is important to acknowledge the limitations of this strategy. As a systemically administered CSF1R inhibitor, PLX5622 affects not only central microglia but also reduces macrophage populations in peripheral tissues, potentially impacting overall immune homeostasis. Furthermore, studies suggest that PLX5622 may act on non-immune cells; for instance, it can alter cholesterol metabolism in brain endothelial cells, which may represent an off-target effect [43]. More importantly, microglia serve as essential immune sentinels in the central nervous system, playing indispensable roles in synaptic pruning, circuit homeostasis, and defense against pathogens. Research confirms that even localized or transient microglial loss can disrupt normal neural function. For example, it impairs nocturnal vigilance in mice, potentially due to disrupted neuron-microglia communication and altered excitability in relevant nuclei, Additionally, microglial depletion compromises the brain’s defense against infections, leading to diminished immune responses, delayed recovery, and increased mortality [44, 45]. Therefore, complete or prolonged microglial depletion may carry unpredictable neurological and systemic risks. Notably, recent studies indicate that a moderate, rather than complete, reduction in microglia (e.g., 50–60% depletion) can also induce a therapeutic phenotypic reversal [46]. Microglia that repopulate following such partial depletion often exhibit reduced reactivity to inflammatory stimuli and display a phenotype more akin to homeostasis [47].

Another study reported that nanoparticle-delivered PLX5622 could selectively deplete M1-type microglia to reduce inflammation in a spinal cord injury model [48]. These findings suggest that future therapeutic strategies might shift towards seeking a controlled and reversible modulation of the microenvironment, rather than mere cellular ablation. This approach aims to achieve therapeutic efficacy while maximally preserving the vital physiological functions of microglia.

It is noteworthy, however, that in clinical practice, CSF1R inhibitors have garnered significant interest for their ability to modulate tumor-associated macrophages, particularly in oncology, where these macrophages often exhibit an immunosuppressive phenotype that promotes tumor progression. In fact, the FDA approved PLX3397 (pexidartinib) in 2019 for the treatment of tenosynovial giant cell tumor, and research in this field continues [49, 50]. However, these studies have not reported the effects of CSF1R inhibitor therapy on human brain microglia. A clinical trial investigating PLX3397 in patients with recurrent glioblastoma did not show efficacy, despite the compound’s good brain penetrance. Histological analysis of resected tumor samples in that study revealed limited to no observable impact on microglia [51]. Therefore, employing microglial depletion methods to treat related diseases still faces significant challenges in clinical translation.

The MAPK pathway is a key regulator of pro-inflammatory cytokine expression. Preclinical studies robustly support its role in pain pathogenesis, as evidenced by the efficacy of various MAPK inhibitors in alleviating hyperalgesia across multiple animal models. For instance, JNK inhibitors reduce osteoarthritic pain in mice and hyperalgesia in chronic constriction injury (CCI) model rats by suppressing inflammatory cytokines [52], ERK inhibitors alleviate peripheral neuroinflammatory pain through downregulation of Nav1.8/Nav1.9 expression [53], and intrathecal administration of p38 inhibitors significantly mitigates both peripheral inflammatory and neuropathic pain [54].

Despite this compelling preclinical evidence, the translation of MAPK pathway inhibitors into effective clinical therapies for chronic pain has been largely unsuccessful. This stark disconnect stems from a fundamental pharmacological challenge: the core components of the MAPK cascade (ERK, p38, JNK) are ubiquitously expressed and vital for basic cellular functions across nearly all organ systems. Consequently, systemic inhibition inevitably disrupts essential physiological processes in off-target tissues, leading to severe dose-limiting toxicities—such as hepatotoxicity, dermatologic reactions, and immunosuppression—that have halted the clinical development of numerous candidates. The case of the ERK inhibitor Mirdametinibhich, shows limited analgesic potential only within specific oncology protocols [55], underscores this predicament; its use is tolerated where the risk-benefit calculus is dominated by life-threatening disease, a context entirely different from managing chronic non-malignant pain. This highlights a critical bottleneck in current analgesic strategy: the inability to precisely target pathological signaling within the complex nervous system while avoiding systemic toxicity.

In this context, our PS NP-induced pain model provides a critical tool to address this translational hurdle. We demonstrate that PS NPs induce pain hypersensitivity not only by activating the spinal MAPK pathway but also by altering the local inflammatory milieu, as recapitulated by the intrathecal injection model. The reversal of hyperalgesia by intrathecal MAPK inhibitors underscores that the maintenance of this pain state is critically dependent on spinal MAPK activation (Figure S11). This finding is pivotal because it re-frames the problem. Instead of the intractable challenge of *systemically* inhibiting a ubiquitous pathway, our model reveals a spatially confined pathology. By mapping how a diffuse exposure leads to MAPK activation in a specific neuroanatomical site, it encourages the rational design of interventions with spatial or cellular selectivity, such as localized intrathecal delivery or the development of nanocarriers that target specific spinal cord cell populations (e.g., microglia) to enhance therapeutic precision and safety [48].

Notably, the potential of PS NPs to engage with intracellular proteins is a critical consideration, as numerous reports indicate that PS NPs can interact with diverse proteins—including TDP-43, hemoglobin, ubiquitin, β-amyloid fibrils, and various enzymes—altering their structure and function [56]. Our study provides a specific example within a defined pathway: we demonstrate that PS NPs physically interact with ERK, JNK, and p38 proteins. This interaction may be facilitated, in part, by the high structural similarity shared by these kinases, particularly in their conserved core phosphorylation motifs. Molecular dynamics simulations suggest that PS NP binding could alter the flexibility of key amino acid residues, potentially enhancing the exposure and recognition of these phosphorylation sites (Thr-X-Tyr) by upstream kinases during physiological processes, thereby facilitating pathway activation.

### Limitations

A key limitation of this study is the lack of direct biomonitoring data on the internal exposure levels of workers to PS NPs, such as concentrations of plastic polymers or their metabolites in blood or urine. Consequently, the reported association is based on presumed exposure within the work environment rather than quantified individual exposure doses. Despite collecting basic information through questionnaires, we were unable to adequately quantify or adjust for all potential confounding factors, including but not limited to exposure to organic solvents, smoking history, other occupational fumes, ergonomic stress, lifestyle differences, and psychological stress. Due to these limitations, this cross‑sectional study cannot establish a causal relationship between PS NP exposure and pain symptoms, and the observed association should be interpreted as preliminary and suggestive.

In addition, the study has several other important limitations. First, it primarily focuses on microglial mechanisms, while the direct effects of PS NPs on other neural cell types—especially neurons—and their complex interactions within pain circuits remain to be fully elucidated. Second, although pull‑down assays provide support for the proposed interaction between PS NPs and MAPK proteins, this mechanism requires further experimental validation. In particular, the specific binding sites suggested by molecular dynamics simulations and their functional consequences on phosphorylation dynamics need to be confirmed through direct approaches such as site‑directed mutagenesis. In addition, the precise mechanistic basis accounting for PS NP-elicited hyperalgesia in a sex-dependent manner awaits further investigation.

Therefore, the current human data primarily serve a hypothesis‑generating function. They strongly indicate the necessity for future prospective cohort studies that integrate individual biomonitoring with clinical pain assessments, while systematically collecting and controlling for a wide range of lifestyle and occupational confounders. Only through such a design can the potential impact of occupational microplastic exposure on neurological health be more reliably evaluated.

## Conclusion

Our study for the first-time link PS NPs to hyperalgesia and reveals the mechanism by which PS NPs induces hyperalgesia in rodents. At the human level, we found that workers in polystyrene production workshops exhibit abnormal pain perception and emotional feedback to pain. Such abnormal pain perception showed a positive correlation with their working years in the industry, suggesting the possible need for improved protective measures in industrial production. In rodent models, short-term PS NPs exposure induced hyperalgesia in mice. The main mechanistic is as follows: Microglia in the spinal dorsal horn internalized a fraction of the PS NPs. Subsequently, PS NPs bound to MAPK pathway proteins in microglia, suggesting that this interaction may induce conformational changes that expose the Thr-X-Tyr phosphorylation sites, facilitating their activation by upstream kinases. Consequentially, the inflammatory milieu in the spinal dorsal horn changed, and neuronal excitability increased. Notably, this hyperalgesia mechanism showed high consistency with other pain models, suggesting it could serve as a novel pain model.

## Supplementary Information

Below is the link to the electronic supplementary material.

Supplementary Material 1

## Data Availability

Data is provided within the manuscript or supplementary information files.

## Declarations

### Note

All experimental protocols involving mice were approved by the Animal Care and Use Committee of Central South University (CSU-2023-0121).
