# Supplementary material for: High-dose polystyrene nanoparticles trigger aberrant activation of the MAPK pathway in spinal cord and pain hypersensitivity
Source: J Nanobiotechnology. 2026 Mar 2;24:322. doi: 10.1186/s12951-026-04186-8 (PMC13059191; doi:10.1186/s12951-026-04186-8)
Supplement: Supplementary file 1 — Supplementary Material 1 [file 12951_2026_4186_MOESM1_ESM.docx]

**Supporting Information**

**High-dose polystyrene nanoparticles trigger aberrant activation of the MAPK pathway in spinal cord and pain hypersensitivity**

Yuan Yin^1, 2, #^ ,Panyang Gu^1, 2，#^, Hanyu Jiang^1^, Yumei Yang^1, 2^, Shujun Wang^1, 2^ Fei Yuan^1, 2^, Wenrui zhong^1, 2^, Miao Chen^1, *^, Meichun Deng^1, 2, *^

**Author information:**

**^1^**Department of Biochemistry and Molecular Biology & Hunan Province Key Laboratory of Basic and Applied Hematology, School of Life Sciences, Central South University, Changsha, 410013, Hunan, China.

**^2^**Hunan Key Laboratory of Animal Models for Human Diseases, Hunan Key Laboratory of Medical Genetics, School of Life Sciences, Central South University, Changsha, China.

**^#^**These authors contributed equally to this work.

**^*^Correspondence**: Meichun Deng, Email: dengmch@csu.edu.cn; Miao Chen, Email: chemcm@csu.edu.cn

**1.Table of contents：**

1. Supplementary Results
2. Supplementary Figures (Figures S1-S12)
3. Supplementary Tables (Tables S1-S3)

**2. Supplementary Results**

**2.1 Characterization of PS NPs and F-PS NPs**

The SEM images (**Figure S1A and S1B**) clearly display the morphologies of PS NPs and F-PS NPs. Both types of nanoparticles exhibit a spherical shape with a relatively uniform size distribution at the nanoscale, as indicated by the 50 nm scale bar. The particle size distribution (**Figure SS1C**) shows that the average diameter of PS NPs and F-PS NPs dispersed in ultrapure water were approximately 49 and 51 nm, respectively. The zeta potential measurement (**Figure S1D**) demonstrated that PS NPs have a zeta potential of -30.5 mV, while F-PS NPs have a value of -19.8 mV. The negative zeta potential values for both indicate that the nanoparticles are electrostatically stabilized in solution, reducing the likelihood of aggregation. F-PS NPs were prepared by swelling with Nile red. The less negative value for F-PS NPs might suggest that the functionalization of Nile red may carry certain charges or interact with the negatively charged groups on the surface of PS during the swelling process.

FT-IR spectra of PS NPs (**Figure S1E**) shows characteristic absorption peaks corresponding to the chemical bonds present in the polystyrene structure, such as the characteristic peaks of C-H stretching vibration of the aromatic ring (3065 and 3026 cm^-1^), C-H_2_ stretching vibration of the main chain (2925 and 2852 cm^-1^), C=C stretching vibration of the aromatic ring (1601, 1493, and 1450 cm^-1^) and C-H bending vibration of the aromatic ring (755 and 697 cm^-1^). The Raman spectra of PS NPs (**Figure S1F**) demonstrated distinct vibrational features. Specifically, the peak located at 618 cm^-1^ was attributed to the C-C-C deformation mode. The peak at 997 cm^-1^ was attributed to the ring breathing vibration. The peak at 1026 cm^-1^ was assigned to the C-H rocking. Moreover, the peak at 1595 cm^-1^ was identified as corresponding to the C-C stretching vibration.

As shown in **Figure S1G**, the F-PS NPs exhibit a distinct fluorescence emission peak, while the PS NPs have a very weak fluorescence signal, which indicates that the functionalization process has successfully introduced fluorescent moieties into the F-PS NPs. The stability of the F-PS NPs over time is investigated by measuring the fluorescence intensity at different time intervals (**Figure S1H**). The results show that the fluorescence intensity remains relatively stable even after 7 days, suggesting good long-term stability of the F-PS NPs in solution.

**2.2 Quantitative detection of PS NPs in mouse central nervous system**

The Raman spectra of F-PS NPs, PS NPs, spinal cord tissue, and brain tissue were obtained, as depicted in **Figure S2**. The Raman spectra of spinal cord and brain tissues were also presented, showing distinct intensity patterns compared to those of F-PS NPs and PS NPs. Notably, the Raman signals of brain and spinal cord tissues did not interfere with the Raman signals of PS NPs, as indicated by the clear separation and non-overlapping of the characteristic peaks. It suggests that the inherent components and molecular vibrations within brain and spinal cord tissues do not mask or distort the characteristic Raman peaks of PS NPs, enabling reliable identification and analysis of PS NPs in these complex biological matrices.

The Raman spectra of blank brain tissue spiked with varying concentrations of F-PS NPs standards (**Figure S3A**) clearly demonstrated a concentration dependent response. As the amount of F-PS NPs added to the blank brain samples increased from 7.5 μg to 1000 μg (curves a-g), a noticeable increase in the intensity of the characteristic peak at 997 cm^-1^ was observed. The peak at 997 cm^-1^ was characteristic of the aromatic ring structure within the F-PS NPs and its intensity change indicated that Raman spectroscopy is highly sensitive to the presence and concentration of F-PS NPs within the mouse brain tissue. The consistent trend in spectral changes with increasing F-PS NPs concentration provides a solid foundation for quantitative analysis. A plot of intensity versus F-PS NPs concentration at 997 cm^-1^ (**Figure S3B**) showed a high determination coefficient (R^2^ = 0.987), suggesting a strong and reliable correlation between the F-PS NPs concentration and the Raman intensity. This linear relationship allows for the accurate quantification of F-PS NPs in unknown samples by simply measuring the Raman intensity at 997 cm^-1^ and applying the calibration equation. In addition, the SERS spectra of 20 points were randomly collected from the blank brain + 75 μg F-PS NPs (**Figure S3C**), and the RSD of the SERS peak intensities at 997 cm^-1^ was determined to be 5.25% (**Figure S3D**). The Raman spectra of six samples from the experimental group of mice (**Figure S3E**) showed variations in the intensity of the 997 cm^-1^ peak, reflecting the different levels of F-PS NPs present in each sample. The magnified view of the 997 cm^-1^ peak in **Figure S3F** further highlights the spectral details of the brain samples. By using the calibration curve established earlier, the F-PS NPs content in each actual brain sample was calculated based on the measured Raman intensity at 997 cm^-1^. The quantitative concentration of F-PS NPs of six brain samples was 283.84, 105.89, 142.52, 211.78, 80.81, 106.29 μg/g, respectively (as listed in **Table S2**).

**2.3. Quantitative detection of PS NP in mouse central nervous system**

Similar to the findings in mouse brain tissue, the Raman spectra of blank spinal cord tissue spiked with different amounts of F-PS standards (**Figure S4A**) also exhibited a concentration dependent behavior. As the quantity of F-PS added to the blank spinal cord samples increased from the lowest to the highest concentration, a linear regression analysis of the Raman intensity at 997 cm^-1^ against the F-PS concentration (**Figure S4B**) yielded a high determination coefficient (R^2^ = 0.996). To assess the reproducibility of the measurement, 20 random Raman spectra were collected from the blank spinal cord + 50 μg F-PS group (**Figure S4C**). The RSD of the characteristic peak intensities at 997 cm^-1^ was calculated to be 6.04% (**Figure S4D**). The Raman spectra of six spinal cord samples from the experimental group of mice (**Figure S4E**) showed that the intensities of the 997 cm^-1^ peak in these samples were relatively similar, indicating comparable levels of F-PS accumulation in each sample. The magnified view of the 997 cm^-1^ peak in **Figure S4F** further revealed the spectral details. Using the established calibration curve, the F-PS content in each spinal cord sample was calculated. The quantitative concentrations of F-PS in the six spinal cord samples were 460.96, 495.21, 484.93, 417.12, 384.93, 313.01 μg/g, respectively (as listed in **Table S3**).

**2.3. Organ-specific distribution of PS NPs in mouse**

In order to investigate the biodistribution of PS NPs in various murine organs, in vivo imaging was conducted by using F-PS NPs. In vivo imaging revealed systemic distribution of F-PS NPs across superficial regions of mouse body surfaces. Subsequent fluorescence imaging of dissected organs demonstrated F-PS NPs accumulation in the brain, spinal cord, liver, spleen, kidneys, lungs, testes, and serum, whereas no detectable fluorescence was observed in cardiac tissues **(Figure S5)**

**2.4 PS NPs induce hyperalgesia in mice**

We established a pilot mouse model. Compared with intragastric administration, intraperitoneal (i.p.) injection demonstrated superior drug bioavailability. In preliminary studies, we administered PS NPs via i.p. injection at varying concentrations (0–50 mg/kg, i.p., n = 3) over a 15-day period **(Figure S6A)** to observe its effects on nociceptive behavior. Both the mechanical paw withdrawal threshold (PWT) and thermal paw withdrawal latency (PWL) showed a dose-dependent reduction **(Figure S6B-D)**.

**2.5** **Microglia depletion rescues PS NPs-induced anxiety-depression-like behavior in mice**

Previous studies report that PS-NPs exposure induces anxiety- and depression-like behaviors in mice. However, the effects of pharmacological microglial depletion on these behaviors remain unknown. Microglial depletion significantly alleviated PS-NPs-induced hyperalgesia, we examined its impact on anxiety/depression-like behaviors. Open field testing showed that partial microglial depletion prevented the PS-NPs-induced reduction in movement distance and time spent in the central area **(Figure S9A-C)**, though it did not significantly affect total locomotion **(Figure S9D)**. Similarly, microglial depletion blocked the PS-NPs-induced increase in immobility time during tail suspension tests **(Figure S9E-F)**. In the elevated plus maze, microglial depletion rescued the PS-NPs-triggered declines in both distance traveled and time spent in open arms **(Figure S9G-I)**.

**2.6 PS NPs induce a reactive state in BV-2 microglia through the MAPK pathway**

Consistent with the in vivo findings in mice, PS NPs significantly promoted the inflammatory proliferation of BV-2 cells in vitro, with the maximal pro-proliferative effect observed at a concentration of 50 μg/mL (**Figure S10A**). Notably, the IC_50_ value of 50 nm PS NPs in BV-2 cells was approximately 215.7 μg/mL (**Figure S10B**). Furthermore, qRT-PCR analysis of inflammatory cytokine expression revealed that PS NPs markedly enhanced the mRNA levels of IL-1β, IL-6, and TNF-α in BV-2 cells (**Figure S10C–E**). Immunofluorescence staining demonstrated that PS NPs treatment induced distinct M1 polarization characteristics in BV-2 cells (**Figure S12)**, including enlarged cell bodies and a morphological shift from a resting spherical shape to an amoeboid-like phenotype (**Figure S10G–J**). F-PS NPs was used to confirm the successful internalization by BV-2 cells and validate the cellular uptake of PS NPs (**Figure S10F**). Importantly, in alignment with the tissue-level observations, PS NPs treatment significantly elevated the phosphorylation levels of ERK, JNK, and P38 in BV-2 cells (**Figure S10K–N**).

**3. Supplementary Figures**


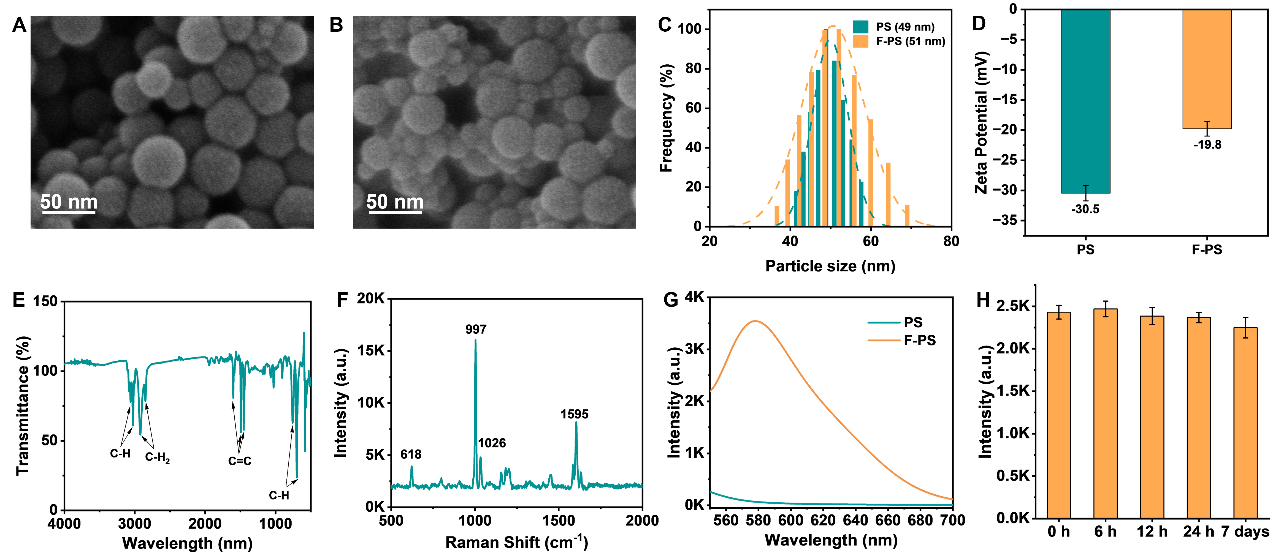


**Figure S1**. SEM images of (A) PS NPs and (B) F-PS NPs. The scale bar is 50 nm; (C) The size distribution of PS NPs and F-PS NPs; (D) Zeta potential values of PS NPs and F-PS NPs; (E) FT-IR spectra of PS NPs presenting characteristic absorption peaks related to the polystyrene structure; (F) Raman spectra of PS NPs; (G) Fluorescence spectra of PS NPs and F-PS NPs; (H) Stability of F-PS NPs over time, measured by fluorescence intensity at different time intervals (0 h, 6 h, 12 h, 24 h, and 7 days), showing relatively stable fluorescence intensity.


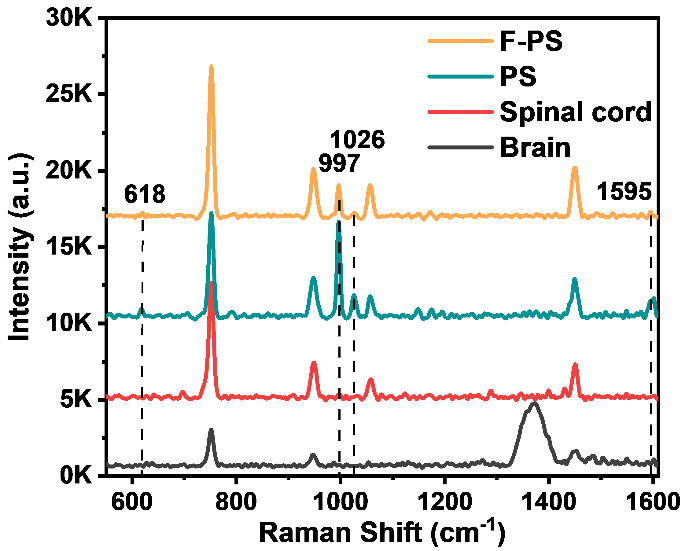


**Figure S2**. Raman spectra of F-PS, PS, spinal cord tissue, and brain tissue.


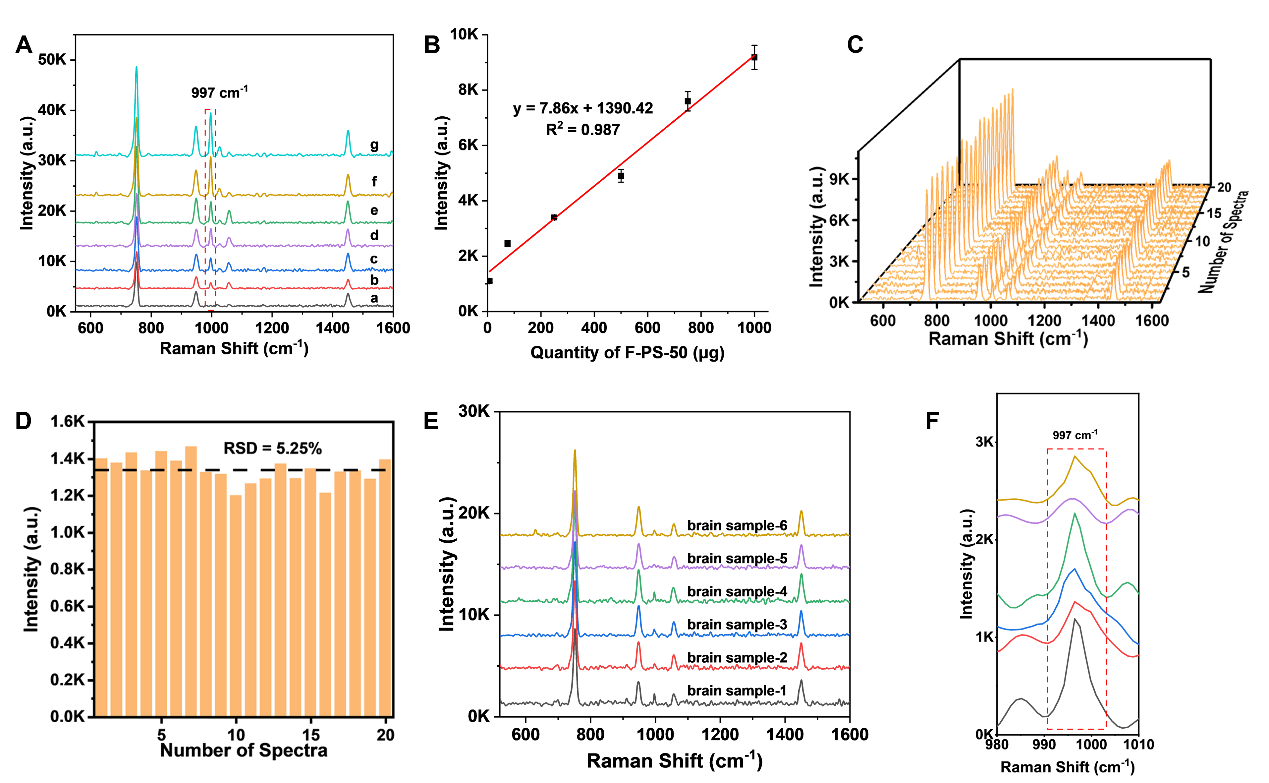


**Figure S3**. (A) Raman spectra of blank brain tissue spiked with different amounts of F-PS standards: (a) blank brain; (b) blank brain +7.5 μg F-PS; (c) blank brain +75 μg F-PS; (d) blank brain +250 μg F-PS; (e) blank brain + 500 μg F-PS; (f) blank brain + 750 μg F-PS; (g) blank brain +1000 μg F-PS. (B) The linear fitting curve of Raman intensity at 997 cm^-1^ for spiked samples; (C) Raman spectra of 20 points randomly selected from the blank brain +75 μg F-PS group and (D) the RSD value of the characteristic peak intensities at 997 cm^-1^; (E) Raman spectra of six brain samples from the experimental group of mice; (F) Magnified view of the 997 cm^-1^ peak in the Raman spectra of the six brain samples.


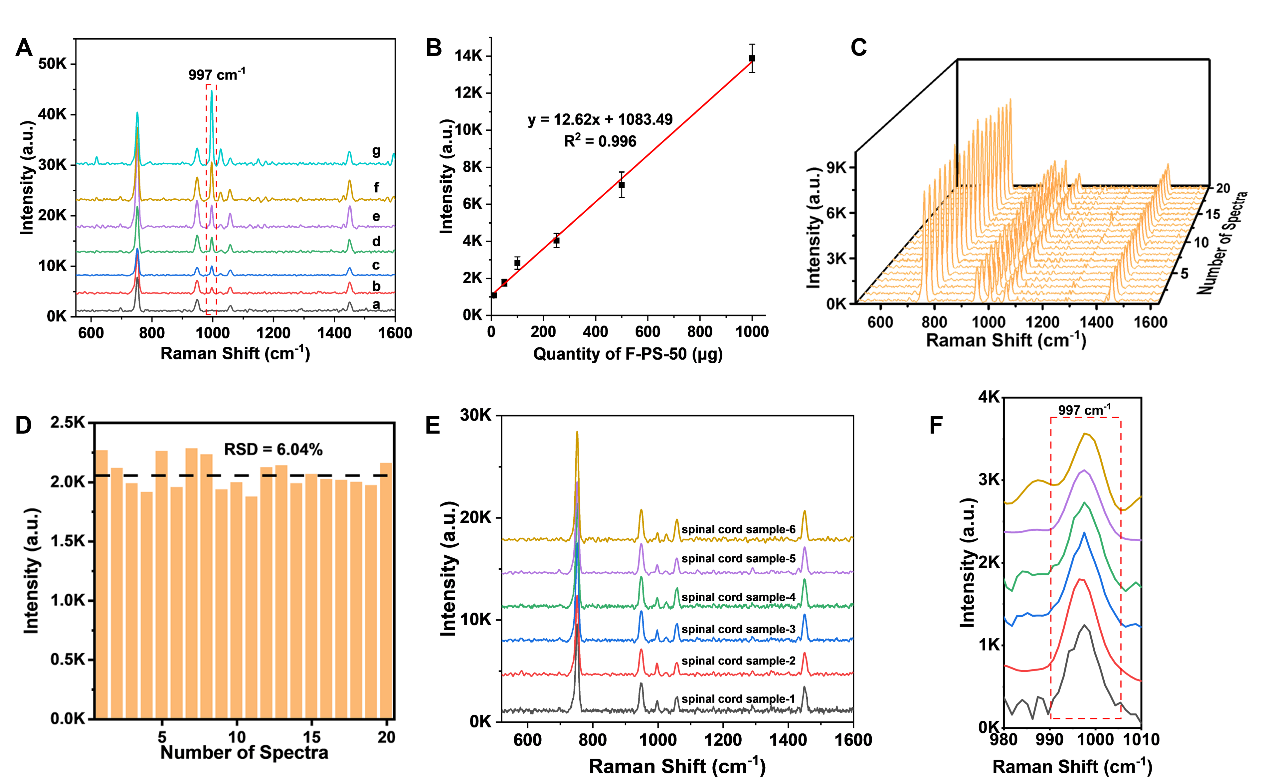


**Figure S4**. (A) Raman spectra of blank spinal cord tissue spiked with different amounts of F-PS standards: (a) blank spinal cord; (b) blank spinal cord + 10 μg F-PS; (c) blank spinal cord + 50 μg F-PS; (d) blank spinal cord + 100 μg F-PS; (e) blank spinal cord + 250 μg F-PS; (f) blank spinal cord + 500 μg F-PS; (g) blank spinal cord + 1000 μg F-PS. (B) The linear fitting curve of the Raman intensity at 997 cm⁻¹ for spiked samples. (C) Raman spectra of 20 points randomly selected from the blank spinal cord + 50 μg F-PS group and (D) the RSD value of the characteristic peak intensities at 997 cm^-1^. (E) Raman spectra of six spinal cord samples from the experimental group of mice. (F) Magnified view of the 997 cm^-1^ peak in the Raman spectra of the six spinal cord samples.

**
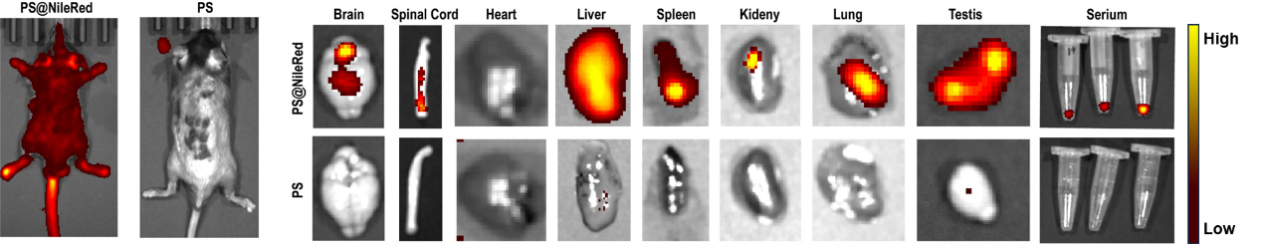
**

**Figure S5.** In vivo imaging results of F-PS NPs in mice.


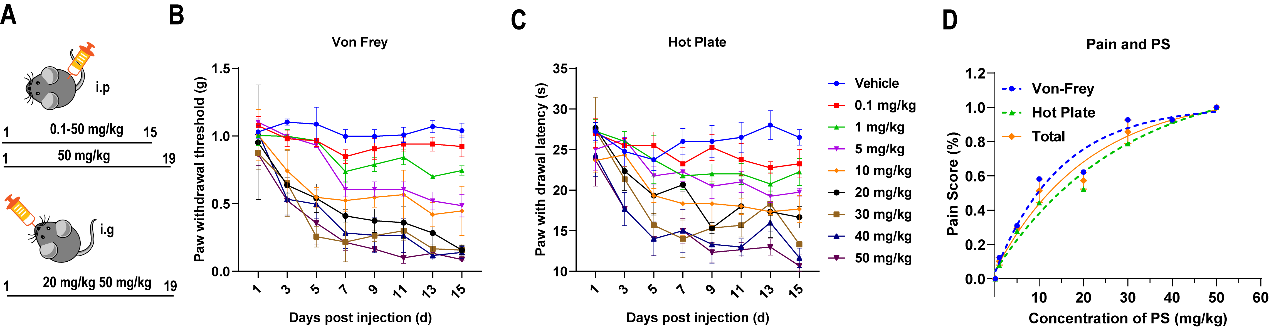


**Figure S6. Effects of intraperitoneal injection of different concentrations of PS NPs on allodynia in mice. (**A) Schematic diagram of intraperitoneal and intragastric administration (n=3-4); (B) Effects of different concentrations of PS NPs on paw withdrawal threshold (PWT) in mice (n=3-4); (C) Effects of different concentrations of PS NPs on paw withdrawal latency (PWL) in mice; (D) Concentration-response curve for allodynia activation.


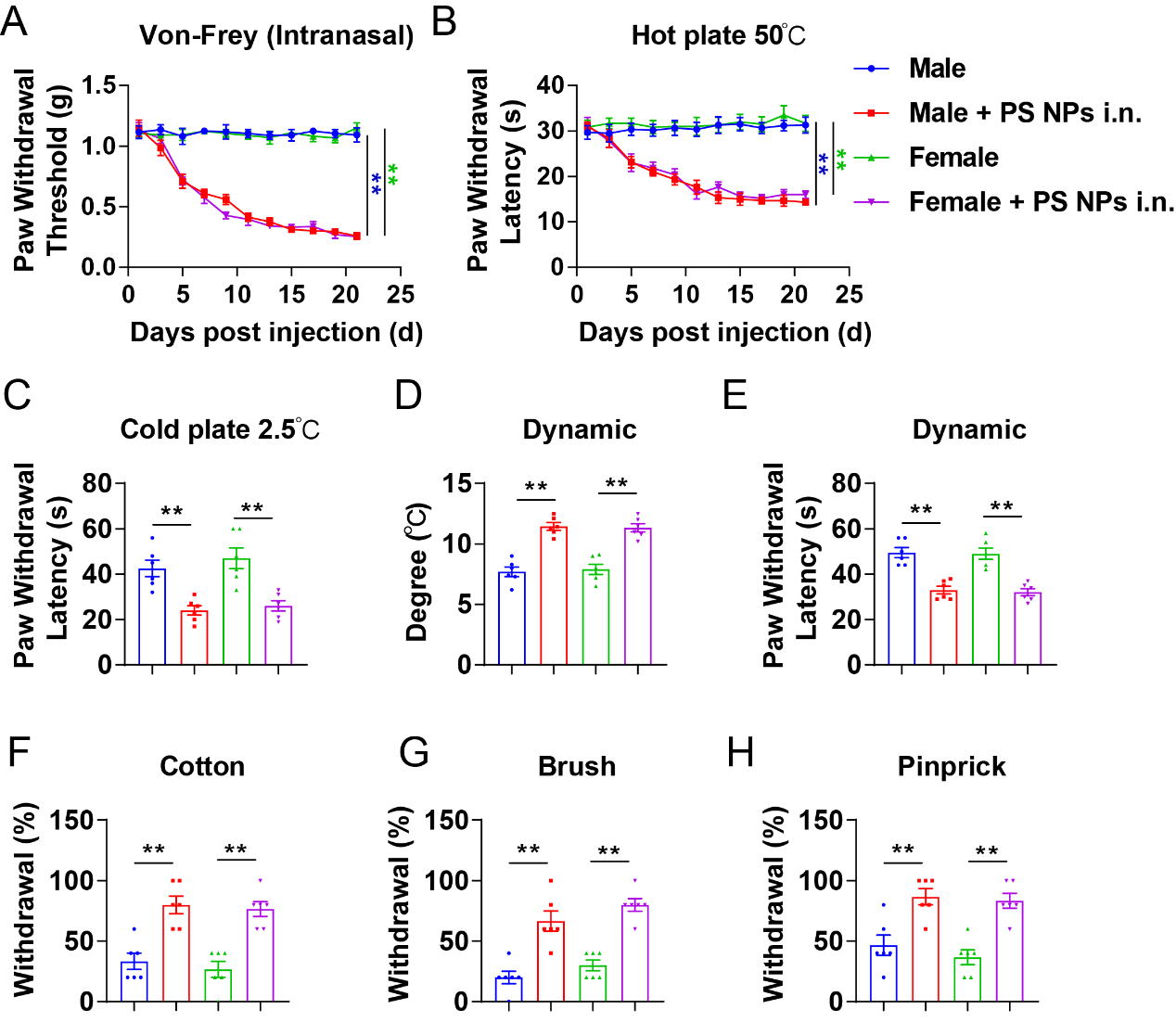


**Figure S7. Intranasal PS NPs-induced pain hypersensitivity is sex-independent in mice.** (A) Mechanical paw withdrawal thresholds, measured by the von Frey test, over time post-administration in Male, Male + Intranasal PS NPs, Female, and Female + Intranasal PS NPs groups; (B) Thermal paw withdrawal latencies, measured by the 50°C hot plate test, over time post-administration in the indicated groups; (C) Cold paw withdrawal latencies, measured by the 2.5°C cold plate test, in the indicated groups; (D) Temperature index results from the dynamic temperature assay for the indicated groups; (E) Paw withdrawal latencies from the dynamic temperature assay for the indicated groups; (F) Percentage of paw withdrawal responses in the cotton swab test for the indicated groups; (G) Percentage of paw withdrawal responses in the brush test for the indicated groups; (H) Percentage of paw withdrawal responses in the pinprick test for the indicated groups; The sample size for all behavioral tests was n=6. Data are presented as mean ± SEM; **p* < 0.05, ** p < 0.01 vs. the corresponding control group (Male or Female group).


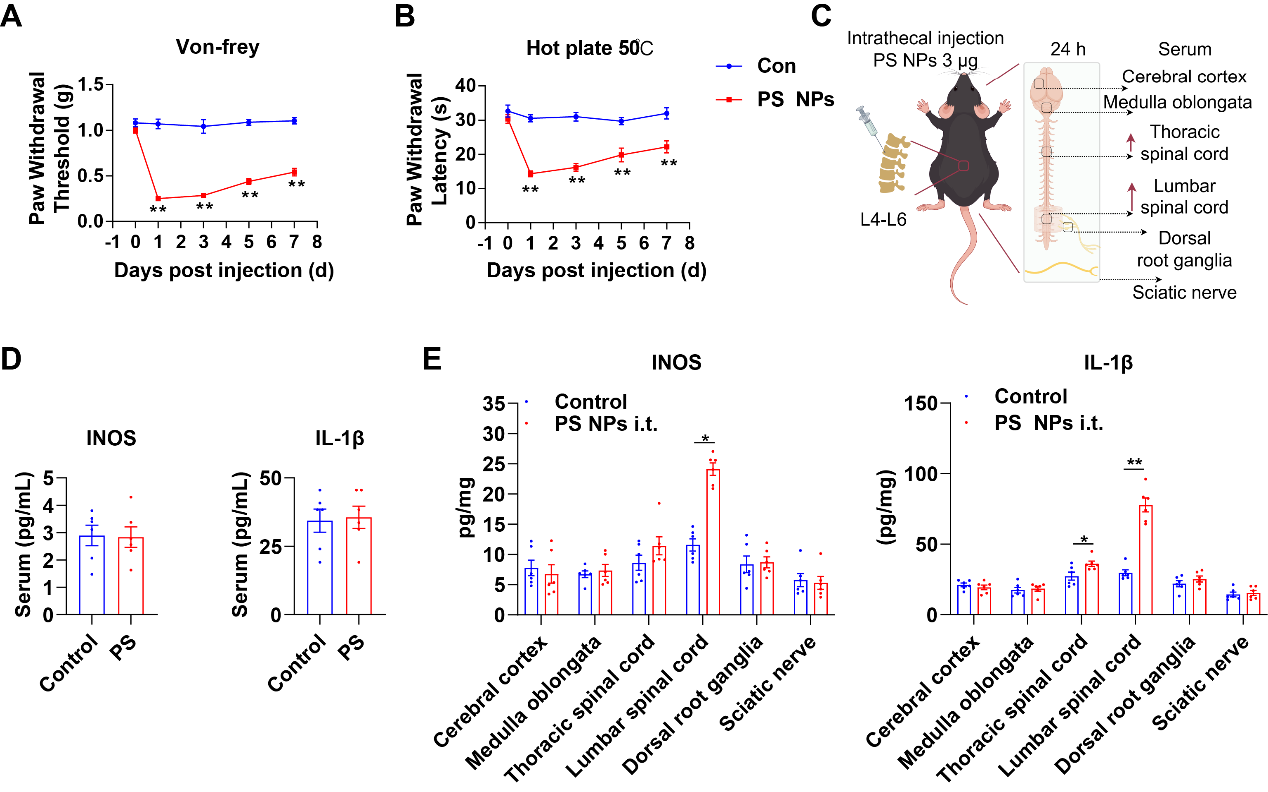


**Figure S8. Intrathecal PS NPs induce rapid changes in the spinal inflammatory milieu.** (A). Assessment of mechanical hypersensitivity (Von Frey test): The paw withdrawal threshold of mice significantly decreased over time following intrathecal PS NPs. injection compared to the control group; (B). Assessment of thermal hypersensitivity (50°C hot plate test): The thermal withdrawal latency of the hind paw was significantly shortened in the PS NP-treated group compared to the control group; (C). Schematic diagram of the experimental procedure and tissue collection: Mice received an intrathecal injection of 3 µg PS NPs at the L4-L6 level. Serum and multiple neural tissues (cerebral cortex, medulla oblongata, thoracic spinal cord, lumbar spinal cord, lumbar dorsal root ganglia, sciatic nerve) were collected 24 hours later for subsequent analysis; (D). Serum inflammatory cytokine levels: The concentrations of inducible nitric oxide synthase (iNOS) and interleukin-1β (IL-1β) in serum were significantly elevated after PS NPs treatment compared to the control group; (E). Inflammatory cytokine levels in various tissues: Following intrathecal PS NPs injection, the concentrations of iNOS (left) and IL-1β (right) were significantly upregulated only in the thoracic and lumbar spinal cord segments. No significant changes were observed in the other examined tissues, including the cerebral cortex, medulla oblongata, lumbar dorsal root ganglia, and sciatic nerve (Data are expressed as mean ± SEM, **P*<0.05, ***P*<0.01 vs. the control group, n=6)


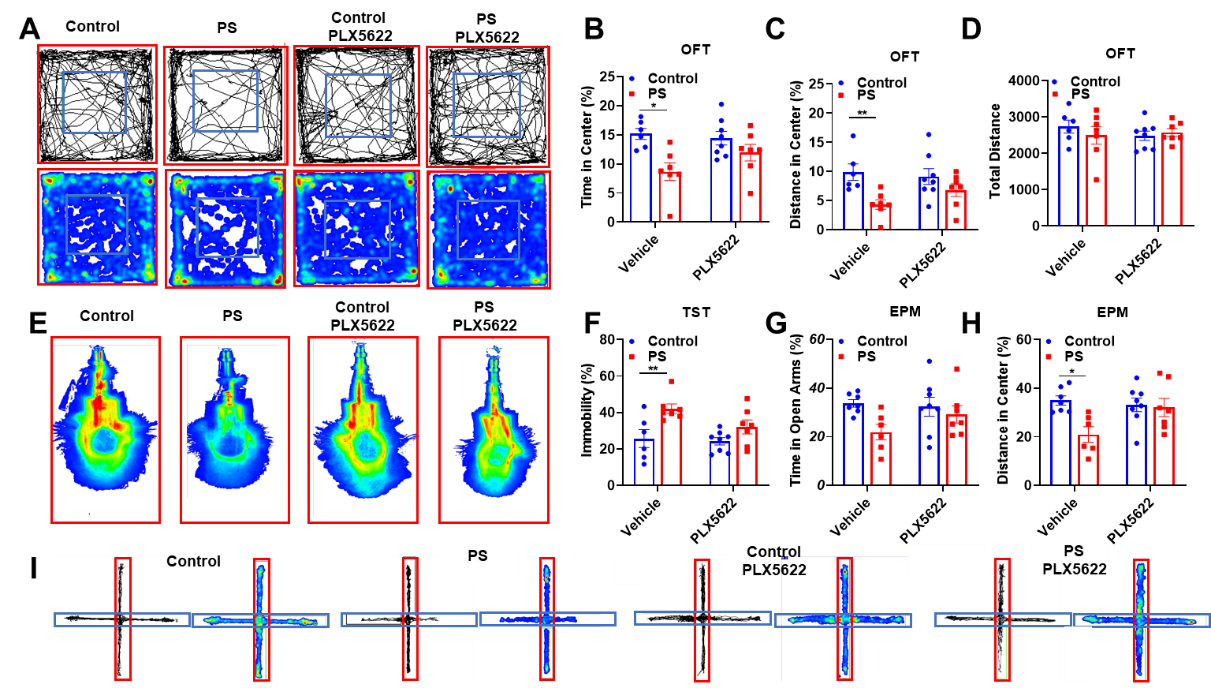


**Figure S9. Microglia depletion rescued the anxiety- and depression-like behaviors induced by PS NPs in mice.** (A). Open field behavior trajectory maps, including trajectory maps and trajectory heat maps. Partial depletion of microglia rescued the central movement distance(B) and central time(C) of PS NPs -treated mice in the open field test, but had no significant effect on the total movement distance(D); (E). Representative heat map of mouse tail suspension test activity(F). Partial depletion of microglia reduced the immobility time of PS NPs -treated mice(G-I); Partial depletion of microglia increased the movement distance and time in the open arms of PS NPs -treated mice in the elevated plus maze test (n=6-7). Data are expressed as mean ± SEM. **p* < 0.05, **p < 0.01 vs. the corresponding control group.


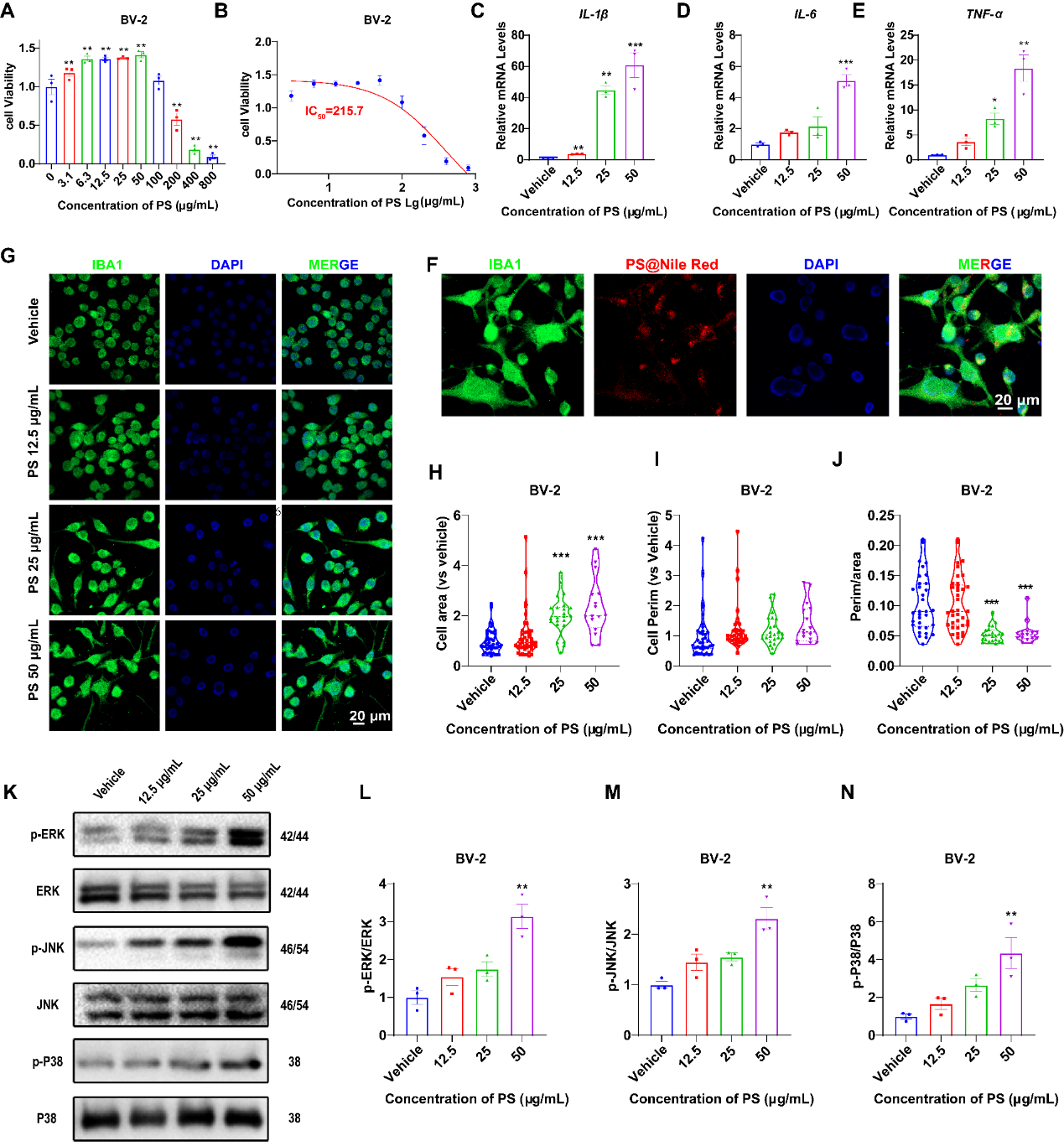
**Figure S10. PS NPs induce a reactive state in BV-2 microglia through the MAPK pathway.** (A) Effect of PS NPs on BV-2 cell viability, with IC_50_ =(B) (*n* = 5); (C-E) PS NPs treatment modulates the expression of inflammatory cytokines in BV-2 cells: interleukin-1β (IL-1β) (C), interleukin-6 (IL-6) (D), and tumor necrosis factor-α (TNF-α) (E) (*n* = 3); (F) Cellular uptake of PS NPs@NileRed in BV-2 cells; (G) Morphological changes in BV-2 cells induced by PS NPs treatment, including cell area (H), cell perimeter (I), and perimeter-to-area ratio (J) (n＞10); (K) Activation of ERK (L), JNK (M), and p38 (N) in BV-2 cells following PS NPs treatment (*n* = 3); Data are presented as mean ± SEM. **p* < 0.05, ***p* < 0.01.


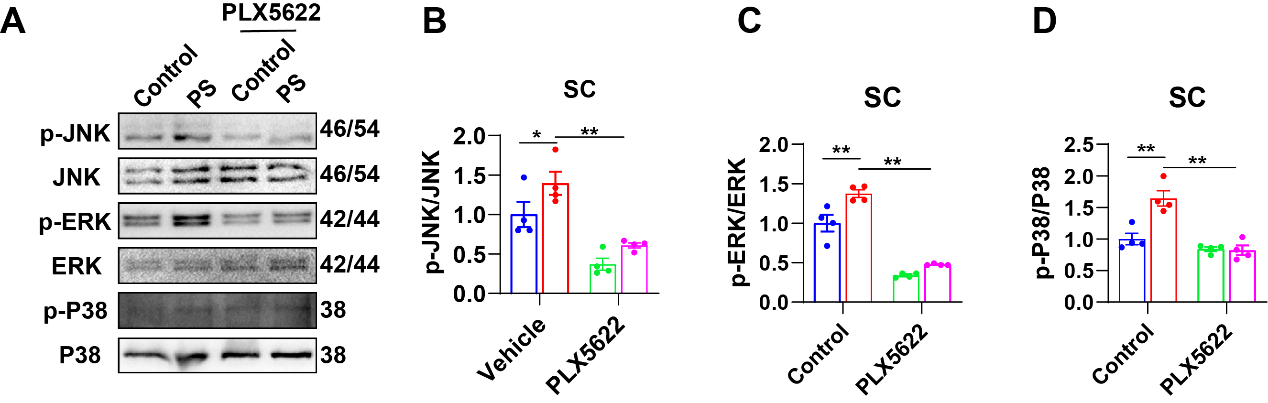


**Figure S11. PLX5622 administration significantly suppresses PS NP-induced MAPK pathway activation in the mouse dorsal horn.** (A) Western blot analysis of MAPK pathway-related proteins in the mouse spinal cord dorsal horn (SC). Targets include phosphorylated JNK (p-JNK), total JNK, phosphorylated ERK (p-ERK), total ERK, phosphorylated p38 (p-p38), and total p38. Groups are: Control (Vehicle or PS NPs intervention) and PLX5622 pretreatment (Vehicle or PS NPs intervention). Numbers on the right indicate the molecular weight (in kDa) of the corresponding proteins;(B) Quantitative analysis of relative p-JNK/JNK expression;(C) Quantitative analysis of relative p-ERK/ERK expression;(D) Quantitative analysis of relative p-p38/p38 expression. **Note:** Dots in different colors represent samples from each group (Blue: Control-Vehicle; Red: Control-PS NPs; Green: PLX5622-Vehicle; Pink: PLX5622-PS NPs). Data are presented as mean ± SEM. **p* < 0.05, ***p* < 0.01, n=4.


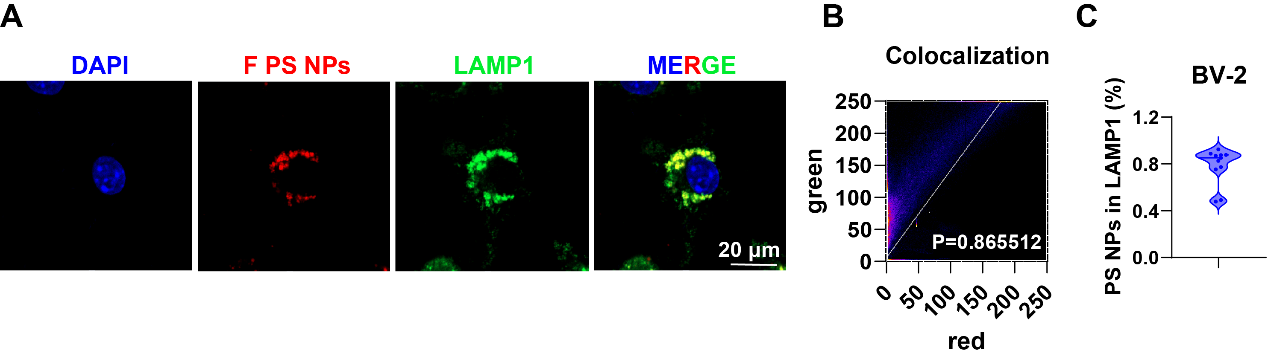
**Figure S12. F-PS NPs are primarily localized within lysosomes of BV-2 cells. (A)** Immunofluorescence colocalization staining of BV-2 cells. The blue channel shows DAPI (nuclei), the red channel shows F-PS NPs, and the green channel shows the lysosomal marker LAMP1. The merged channel (MERGE) reveals significant colocalization (yellow) between the red signal of F-PS NPs and the green signal of LAMP1. Scale bar: 20 µm; **(B)** Scatter plot analysis of colocalization signals. The x-axis represents the red fluorescence intensity of F-PS NPs, and the y-axis represents the green fluorescence intensity of LAMP1. The result indicates a strong colocalization correlation between the two signals (P = 0.865512); **(C)** Quantification of the distribution proportion of F-PS NPs within LAMP1-positive structures in BV-2 cells. The data show that approximately 78% of F-PS NPs are located within LAMP1-marked lysosomal structures(n=10).

1. **Supplementary Tables**

**Table S1** demographics/potential confounding factors

|  | Plastics factory worker (n=180) | General People（n=187） |
| --- | --- | --- |
| Age (years) | 26.63 ± 0.36 | 24.81 ± 0.43 |
| Smoking rate (%) | 32.2% | 27.3% |
| Female proportion (%) | 20% | 45.2% |
| BMI | 21.63 ± 0.36 | 22.16 ± 0.58 |
| Daily working hours (hours) | 8.68 ± 0.07 | 8.56 ± 0.10 |
| Average income (CNY) | 7241.18 ± 189.75 | 4508.47 ± 223.72 |

**Table S2**. Peak intensity at 997 cm^-1^, quantitative concentration of F-PS, and RSD values for six brain samples.

| Sample | Peak intensity at 997 cm^-1^ | Quantitative concentration of F - PS (μg/g) | RSD (%) |
| --- | --- | --- | --- |
| brain sample-1 | 1396 | 283.84 | 4.08 |
| brain sample-2 | 521 | 105.89 | 6.17 |
| brain sample-3 | 701 | 142.52 | 2.89 |
| brain sample-4 | 1042 | 211.78 | 8.60 |
| brain sample-5 | 398 | 80.81 | 3.97 |
| brain sample-6 | 522 | 106.29 | 3.14 |

**Table S3**. Peak intensity at 997 cm^-1^, quantitative concentration of F-PS, and RSD values for six spinal cord samples.

| Sample | Peak intensity at 997 cm^-1^ | Quantitative concentration of F - PS (μg/g) | RSD (%) |
| --- | --- | --- | --- |
| spinal cord sample-1 | 992 | 460.96 | 5.36 |
| spinal cord sample-2 | 1065 | 495.21 | 4.88 |
| spinal cord sample-3 | 1043 | 484.93 | 5.19 |
| spinal cord sample-4 | 898 | 417.12 | 3.24 |
| spinal cord sample-5 | 828 | 384.93 | 6.16 |
| spinal cord sample-6 | 674 | 313.01 | 5.72 |
